# Lessons learned from surveying Galápagos marine iguanas using drones

**DOI:** 10.1186/s12983-026-00623-4

**Published:** 2026-07-04

**Authors:** Andrea Varela-Jaramillo, Christian Winkelmann, Gustavo Pazmiño, Jenifer Suárez-Moncada, Estefanía Altamirano, Eduardo Espinoza, Marjorie Riofrío-Lazo, Diego Páez-Rosas, Gonzalo Rivas-Torres, Juan M. Guayasamin, Sebastian Steinfartz, Amy MacLeod

**Affiliations:** 1 Institute of Biology, Molecular Evolution and Systematics of Animals, University of Leipzig, Leipzig, Saxony Germany; 2 3Diversity, Quito, Pichincha, Ecuador; 3 Eberswalde University for Sustainable Development, Eberswalde, Brandenburg, Germany; 4 Parque Nacional Galápagos PNG, Dirección de Ecosistemas, Isla Santa Cruz, Galápagos, Ecuador; 5 Colegio de Ciencias Biológicas y Ambientales COCIBA, Universidad San Francisco de Quito USFQ, Quito, Pichincha, Ecuador; 6 Galapagos Science Center GSC, USFQ & UNC-Chapel Hill, Isla San Cristóbal, Galápagos, Ecuador; 7 Fundación Conservando Galápagos, Galapagos Conservancy, Isla Santa Cruz, Galápagos, Ecuador; 8 Wildlife Ecology and Conservation, University of Florida, Gainesville, FL USA; 9 Laboratorio de Biología Evolutiva, Instituto Biósfera, Universidad San Francisco de Quito USFQ, Quito, Pichincha, Ecuador; 10 Museum für Naturkunde – Leibniz Institute for Evolution and Biodiversity Science, Berlin, Germany

**Keywords:** Aerial surveys, Monitoring, Photogrammetry, Protocols, Technology, UAVs

## Abstract

**Background:**

Monitoring wildlife in remote areas is a key challenge in conservation, with traditional methods proving increasingly inadequate in the face of accelerating biodiversity loss. Uncrewed Aerial Vehicles (UAVs) or drones help bridge data gaps, but methods require careful development and validation to ensure protocols are appropriate, accessible, reproducible, and generate reliable data. Herein, we develop detailed UAV-based protocols for surveying the endangered marine iguana (*Amblyrhynchus cristatus*), a lizard that is endemic to the coastlines of the Galápagos Islands, Ecuador. We outline steps from image collection and processing, through locating and counting animals, before validating results against traditional methods.

**Results:**

We find that UAV-based surveys outperform traditional ground-based surveys in terms of count reliability and effort in the field in different types of terrain and various population densities. Moreover, we show that consumer-level drones can be used effectively — even by newly trained pilots — and describe a standardised manual flying protocol that mimics automated flying whilst maintaining flexibility in the field. Finally, we recommend the use of orthomosaics (geometrically corrected, high-resolution aerial image maps) for surveys on flat terrains and 3D models (digital representations of the surface in three dimensions) for cliffs and compare several common image-processing platforms in terms of success to reconstruct marine iguanas.

**Conclusion:**

Our protocols advance the effective monitoring of Galápagos marine iguanas. Whilst they were specifically developed for this species, we postulate that these could be applicable for other species across the archipelago, or in coastal and open landscapes worldwide.

**Supplementary Information:**

The online version contains supplementary material available at https://doi.org/10.1186/s12983-026-00623-4.

## Background

The Galápagos Islands are a volcanic archipelago located approximately 1,000 km from continental Ecuador in South America. Its independent origin, constant formation and movement of islands, and particular oceanographic conditions have led to the evolution of numerous endemic and highly unique species [1, 2]. This group of islands contains more than 2,700 native species, including vertebrates, invertebrates, and plants. Of these, 30 to 60% are endemic [3, 4], highlighting the distinctiveness of the archipelago. The International Union for the Conservation of Nature (IUCN) notes that over 200 of these species face some level of threat [5], mainly related to the increasing severity of multiple anthropogenic stressors over recent decades, which has led to significant population declines [6, 7]. Active monitoring of endemic species is therefore essential to ascertain population trajectories and effectively direct conservation actions.

In the Galápagos, representatives of the Galápagos National Park (GNP) — the institution responsible for the management and conservation of this archipelago — coordinate efforts with various actors (such as governmental, academic, non-governmental organisations, and the organised civil society) to generate research, monitoring, and conservation actions [8]. One notable effort is the “emblematic species expedition”. These have been conducted annually since 2014 to monitor the abundance and health status of marine iguanas (*Amblyrhynchus cristatus*), sea turtles (*Chelonia mydas*), and sea lions (*Zalophus wollebaeki*), as well as other select species, environmental conditions like water quality, and plastic pollution [9–12]. Generally, these monitoring expeditions employ traditional methods, which consist of walking surveys on flat terrains performed along defined transects, or viewing coastlines from a moving boat whilst counting individuals sighted. Such monitoring can be time-consuming, expensive, imprecise, and potentially disturbing to wildlife [13], as well as risky for surveyors, since boat landings are made on exposed coastlines where accidents are an ever-present danger. Moreover, data collected may not be readily compared between observers or otherwise verified [14]. For these reasons, adopting modern methods could help to improve the accuracy, efficacy, replicability, and transparency of monitoring efforts.

Drones, also known as Uncrewed Aerial Vehicles (UAVs), are a rapidly advancing technology that has proven helpful for species monitoring [15]. In the Galápagos, UAVs have been used in various projects, from monitoring large-bodied marine species such as sharks [16], mapping vegetation, crops and the spread of invasive species [17, 18], to classifying intertidal habitats and studying volcanic eruptions [19, 20]. While UAV approaches are increasingly incorporated into GNP monitoring campaigns, standardised workflows regarding aerial data collection and analysis are lacking. Therefore, there is an urgent need to develop and formalise clear protocols for UAV-based surveys to ensure that these methods can be applied effectively in future expeditions.

In 2020, the Iguanas from Above project (https://www.iguanasfromabove.com; based at the University of Leipzig in Germany, in collaboration with the Galapagos Science Center in Ecuador) began (i) testing the utility of performing marine iguana monitoring using UAVs, and (ii) assessing different approaches for processing the aerial images collected and detecting and counting the individuals. The marine iguana is an endemic species that occurs on all islands of the Galápagos and comprises 11 subspecies [21], whose conservation statuses vary from Vulnerable to Critically Endangered [22]. Historically, marine iguana censuses have been performed using traditional ground-based methods such as simple counts, and in some cases, capture-mark-resight [23]. However, ground-based surveying has several drawbacks; for instance, surveyors walking nearby can disturb animals, causing them to hide and be excluded from the census. In cases where the density is very high, rough approximations of the number of individuals are typically made. These counts cannot be verified later, and their accurate collection also depends on the surveyor’s expertise. Importantly, a lack of safe access to remote iguana colonies and the resource-intensive nature of these methods mean that traditional approaches have proved inadequate for the challenge of obtaining regular, detailed, and reliable counts over the wide range of the species. This means that new approaches are needed to address this data gap. The overall goal of our project is therefore to obtain a comprehensive and detailed census of Galápagos marine iguanas across their entire range, whilst simultaneously developing a UAV-based survey method for this species. During our project, we conducted a series of tests to develop best-practice techniques for wildlife surveys of the Galápagos coastlines, whilst circumventing particular challenges, such as flying amateur-level drones from moving boats and sometimes using beginner pilots — trained for around one week by our team — before the expedition. These pilots were usually volunteers, not local managers, since at this time, just a few GNP rangers flew drones. A central aim of Iguanas from Above is to share the valuable experience we accrued during this work to facilitate conservation and research using UAVs.

Drones are increasingly used in conservation, and existing studies and protocols already provide detailed information regarding equipment, software, relevant flight parameters, and recommendations for effective species monitoring [15, 24, 25]. Our work began with adapting these methods for the purpose of surveying a unique, highly camouflaged species living in remote and inaccessible habitats. During this process, we identified information not typically found in published protocols or scientific articles. For example, we lacked detailed information for flying drones safely from small boats, an approach which is only briefly described in studies of marine animals [16, 26, 27]. The lack of detail is understandable since the aim of those studies was not to provide a protocol for drone surveys; however, that information is still useful, especially when drones are being used by non-specialists and as part of an expanding conservation toolkit. During our work, we collaborated broadly with other researchers and conservation managers to develop an accessible aerial-based technique for monitoring in the Galápagos; an approach that could be highly applicable to other low-mobility species living in coastal regions, as well as in open landscapes such as deserts and wetlands. In order to contribute more broadly to the application of drones in conservation, we herein describe our protocols and insights, including detailed parameters and methods to facilitate the use of UAVs in other applications.

**Fig. 1:**
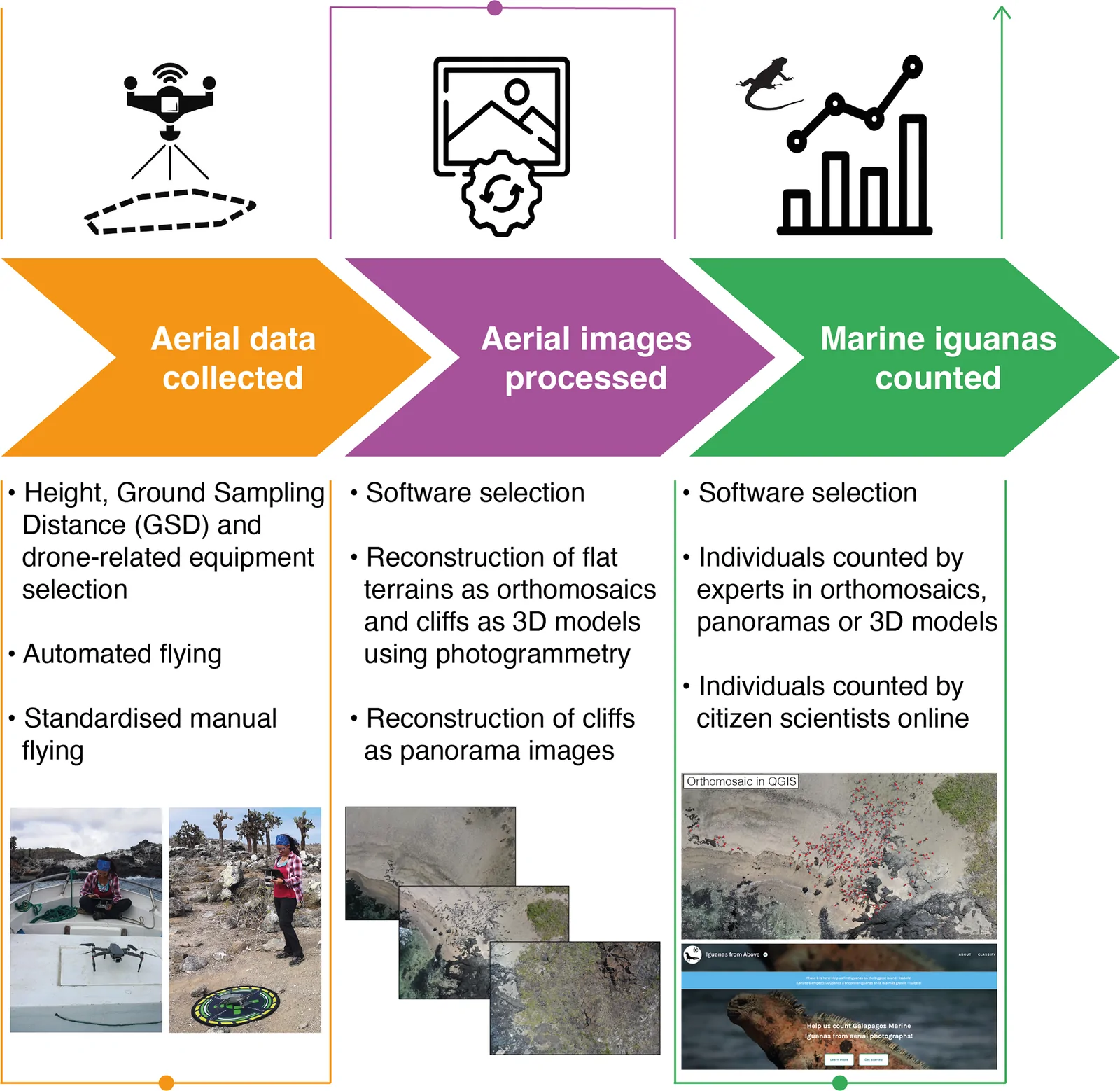
Summary of our workflow for collecting aerial images, processing them, and counting marine iguanas. When collecting high-quality aerial images, it is important to consider parameters such as flight altitude and image resolution in advance. We outline two options for data collection: flying autonomously or manually. For processing the images, the appropriate software depends on whether the terrain being surveyed is flat — in which case orthomosaics are favoured — or steeply inclined, where 3D models or panoramas are more suited. To count iguanas, we have thus far relied on manual counting by experts in GIS software, as well as aggregated counts from online volunteers (citizen science). As the citizen science approach has been discussed elsewhere [28], we focus here on the process of manual counting by experts

This paper, therefore, outlines in detail our workflow, from aerial image collection to analysis and results, including comparisons of different approaches (Fig. 1). Our team of experts and online citizen scientists have conducted marine iguana counts using aerial images. Below, we describe the manual process used by our experts. The citizen science approach used is explained in detail in our previous work [28]. To validate the method, aerial counts of marine iguanas were compared with traditional ground-based surveys undertaken across a variety of terrains and population densities, allowing us to assess the broader applicability of UAV-based monitoring. This expands upon our previous pilot study — which compared ground-based surveys to aerial approaches across four sites, but did not include cliffs or high-density colonies [29] — situations which this work directly addresses.

## Materials and methods

### Description of equipment

**Fig. 2:**
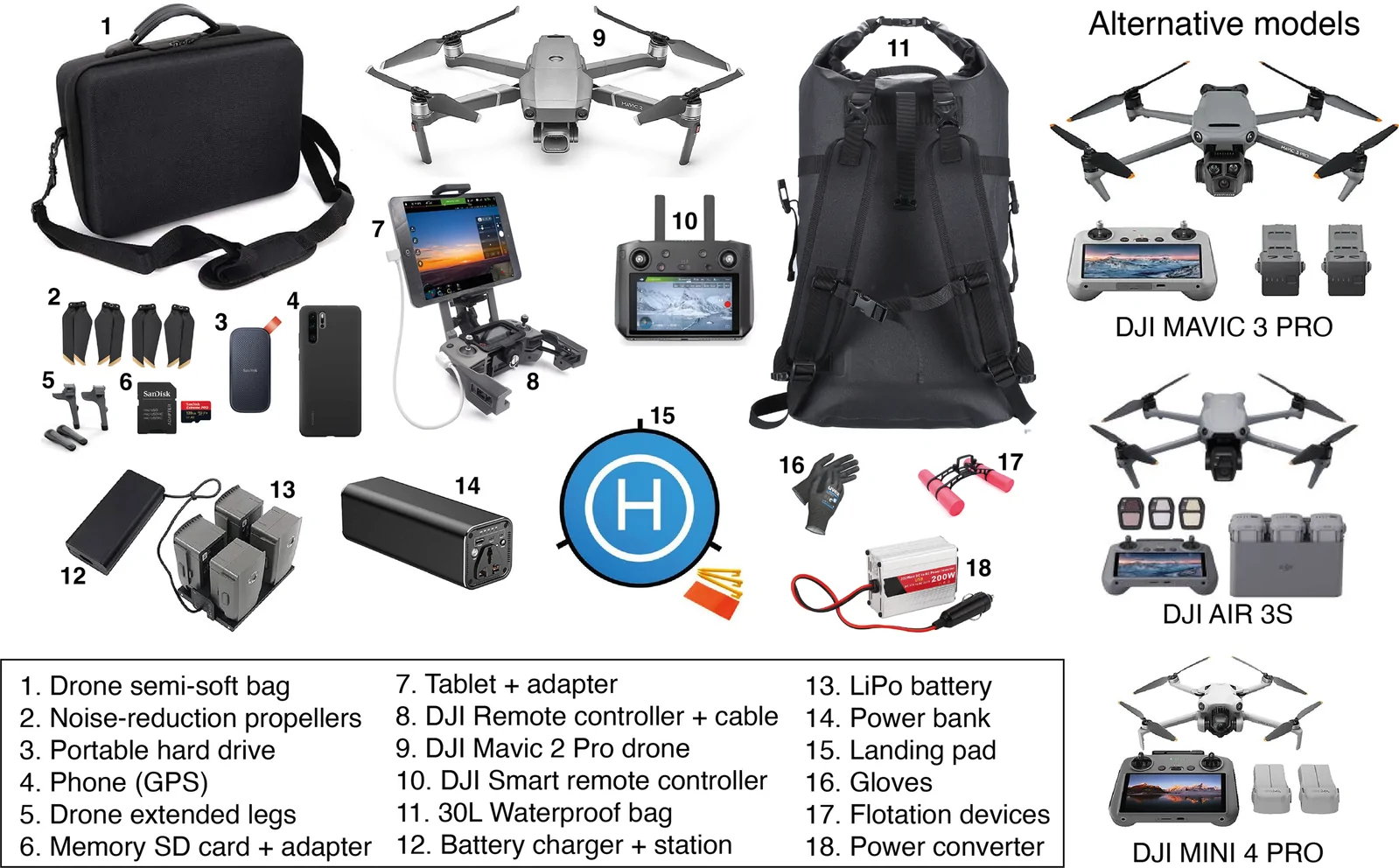
Basic starter kit of equipment recommended for aerial-based monitoring. The kit includes products directly involved in image collection, such as aircraft and remote controls, as well as safety-related equipment, such as landing pads and gloves. Alternative current drone models are also shown, since the DJI Mavic 2 Pro is now discontinued. Drone specifications are explained in the discussion, under limitations of the equipment used

From 2020 to 2023, we conducted annual surveys of marine iguana colonies using UAVs (four fieldwork seasons in total), during the reproductive season (December and January), covering the entire distribution range of the species [30]. Table 1 lists all the UAV-related equipment we have used and integrated over time. The selected products are primarily chosen for their transportability, availability of cameras and sensors, affordability, stability, frequency of use, market availability, and user-friendliness. Figure 2 illustrates the equipment we consider essential to bring into the field. As in many protected regions, drone use in the Galápagos is highly regulated by local authorities; all necessary permits to fly drones in this region were obtained.

**Table 1:** List of equipment used in our UAV-based wildlife monitoring in the Galápagos. Each product includes information on the equipment category, specifications (e.g., flight time and camera specifications for drones), purpose, cost, weight, and current market availability (as of April 2026). Reference prices are in US dollars and are current as of April 2026. (*) Second-hand costs for used, discontinued drone models. (**) Newer alternatives

Category	Product	Key Specifications/ Use	Cost (USD)	Weight (g)	Status
Drones	DJI Mavic Pro	Flight time: 27 min; Camera: 1/2.3”, 12 MP, image resolution at 50 m of 1.5–2 cm/pixel	300*	734	Discontinued
	DJI Mavic 2 Pro	Flight time: 31 min; Camera: Hasselblad 1” CMOS, 20 MP, image resolution at 50 m of 1 cm/pixel	500*	907	Discontinued
	DJI Mavic 2 Enterprise	Flight time: 31 min; Camera: 12 MP, image resolution at 50 m of 1.5–2 cm/pixel	1,800*	1,100	Discontinued
Remote Controllers (RC)	DJI Remote Controller	–	150	316	–
	DJI Smart Controller	–	400	630	Discontinued
Batteries & Charging	LiPo Intelligent Flight Battery (Mavic 2 Pro)	Capacity: 3,850 mAh; Charging temp: 5–40 °C; Max current: 80 W	150	297	–
	Battery Charging Hub	Capacity: 4 batteries (simultaneous charging)	100	–	–
	Xingdoz Power Bank Black-N6	Capacity: 31,200 mAh; 100 W; Charges equipment in the field	100**	898	Discontinued
	Engine Power Converter	Capacity: 300 W	50	–	–
Displays	Apple iPad Pro 10.5” (2019)	Used as a remote screen	200*	469	Discontinued
	Samsung Galaxy Tab Active 2 (2017)	Used as a remote screen	200	415	Discontinued
	Apple iPhone 7 Plus 128 GB	Used as a remote screen	100*	188	Discontinued
	Huawei P30 Pro 128 GB	Used as a remote screen	270	192	–
Data Storage	SanDisk Ultra 64 GB + Adapter	Drone data storage	15	–	–
	SanDisk 1 TB Extreme Portable SSD	High-speed, temporal data storage	150	–	–
Accessories	Extended Legs (Mavic 2 Pro)	Raise the drone clearance on landing	10	–	–
	Landing Pad	Protects the drone from sand/ground	20	–	–
	UAV Flotation Device	Protects the drone from contact with water	25	–	–
	Noise-Reduction Propeller Blades	Reduces animal disturbance	15	–	–
Extra Equipment	30 L Waterproof Dry Bag	Transport equipment in boat/field	30	–	–
	Nylon Work Gloves	For drone launch/recovery	20	–	–

### Initial considerations for using drones

Flight altitude can be a trade-off between the amount of time/effort required to survey a given area (with higher altitudes covering larger areas) and the visibility of target items in the image. To ascertain the appropriate flight altitude, we sought the highest altitude at which the marine iguanas could be reliably seen, and this, in turn, depends on the Ground Sampling Distance (GSD) of the image captured. GSD is the distance between two consecutive pixel centres measured on the ground in an aerial image, typically expressed in cm/pixel. GSD defines the spatial image resolution, where lower values (e.g., 1 cm/pixel) indicate greater detail; see https://support.pix4d.com/hc/en-us/articles/202559809 and [24, 31]. GSD also depends on the camera specifications, such as focal length, sensor width and image dimensions. The Mavic 2 Pro has a 35-mm-equivalent focal length of 28 mm (the actual physical focal length used in GSD calculations is 10.26 mm) and an optical sensor width of 1-inch CMOS (the sensor dimension used in GSD calculations is 13.2 mm) [32]. The actual focal length is the lens’s true physical property, while the equivalent focal length is a reference value scaled to full-frame to make field-of-view comparisons intuitive. The default image dimensions for this model are 5472 × 3648 pixels. A GSD calculator is available at https://support.pix4d.com/hc/en-us/articles/202560249. GSD can also be calculated with:

$$\begin{aligned}&\:\mathrm{G}\mathrm{S}\mathrm{D}\left(\mathrm{c}\mathrm{m}\:\mathrm{p}\mathrm{e}\mathrm{r}\:\mathrm{pixel}\right)=\\&\frac{\mathrm{camera\:sensor\:width}\left(\mathrm{m}\mathrm{m}\right)\times\:\mathrm{altitude}\left(\mathrm{m}\right)\times\:100}{\mathrm{camera\:focal\:length}\left(\mathrm{m}\mathrm{m}\right)\times\:\mathrm{image\:width}\left(\mathrm{pixels}\right)}\end{aligned}$$

The marine iguanas are relatively large for lizards (Snout-vent length, SVL: 12–49 cm [21]) but are typically well camouflaged against the lava rocks, suggesting a high image resolution is needed. Therefore, we aimed to obtain a GSD of 1 cm/pixel or lower. To obtain this, we flew at between 20 and 30 m (in relative height above the starting point) over flat terrain with the camera facing down (-90° camera angle or nadir), depending on the subspecies, which vary markedly in size between islands [21]. For example, we flew at 20 m (GSD = 0.46 cm/pixel) for *Amblyrhynchus cristatus nanus* from Genovesa Island (mean SVL 20 cm, range 14–24), at 25 m (GSD = 0.58 cm/pixel) for *A. cristatus venustissimus* from Española Island (mean SVL: 26 cm, range 20–32), and at 30 m (GSD = 0.69 cm/pixel) for *A. cristatus cristatus* from Isabela Island (mean SVL: 34 cm, range 16–48). When surveying cliffs or areas with steep coastal slopes, we flew at ~ 15 m above the sea, with the camera facing diagonally (-40° camera angle) and maintaining at least 5 m horizontally from the cliff (frontal drone obstacle sensors help to maintain this as a steady distance).

We preferentially launched and followed the drone from a boat; this accelerates the pace of surveys, avoids difficult boat landings, and allows access to areas where landing is impossible due to a lack of safe sites and difficult sea conditions. This generally gave us a reasonable distance from the colony to launch the drone (more than 50 m when flying from a small dinghy and over 200 m when flying from a larger vessel). However, on some occasions, we flew the drone from land. There, we located the colony and launched the drone approximately 30 m away (at this distance, we observed no reaction from the iguanas to the surveyors). We first reached the designated altitude and then approached the animals to avoid any disturbance caused by flying overhead at lower altitudes. During flights, we aimed to survey the entire area where individuals occurred, covering all rocky and sandy areas, as well as nearby vegetation that bordered these zones. We maintained a constant speed of ~ 2 m/s during the entire flight and used noise-reduction propellers to minimise disturbance. We did not measure disturbance directly, but our observations indicated that the animals did not exhibit significant reactions — such as running or fleeing from their resting areas — to the presence of the drones. In a few cases, they looked upwards or, at most, changed their position on the rock.

The drone we selected has a Global Navigation Satellite System (GNSS, GPS+GLONASS, https://www.dji.com/de/mavic-2/info) with a horizontal hovering accuracy of ± 1.5 m when using GPS positioning. Projects requiring more precision, for example, when needing to measure objects or areas, should use an RTK (Real-Time Kinematic) system, which can correct satellite errors in real-time or post-flight. However, it is important to consider that this may involve bringing bulky equipment and having someone on land for up to several minutes — depending on the environment and equipment — to ensure the RTK connects to sufficient satellites to obtain millimetric precision (https://geonadir.com/rtk-explained/). Additionally, Ground Control Points (GCPs) — dispersed throughout the surveyed area and whose coordinates are taken on the ground — could be used to help correct the measurement errors in an orthomosaic during the reconstruction process.

### Testing aerial image collection methods

#### Automated versus manual flying

To generate good-quality maps (orthomosaics) and enable the reliable counting of objects, the UAV flight path must ensure that aerial images collected have enough overlap (70–80%; [33]); this should be fairly straightforward when using an automated flying approach, but is more challenging when undertaking flights manually. Moreover, for the purposes of repeat surveying of sites — especially by less experienced UAV pilots or by multiple people — automated flying is an attractive option since an area can be precisely (re)surveyed with minimal skill. However, for our equipment and software (DJI Mavic 2 Pro + DJI Pilot/Drone Deploy v.4/Map Pilot Lite), this approach typically requires a functional internet connection, which was initially not available in our study area. Furthermore, for species like the marine iguana, which spend a significant amount of time in the intertidal zone, the relevant area for surveying changes with the tide. If the survey timing cannot adapt to the tide — as is often the case for highly remote areas covered by boat-based expeditions — automated flying can result in either missing large areas of relevant coastline (at low tide) or spending valuable time surveying irrelevant areas of water (at high tide). For these reasons, we sought to develop a standardised manual flying protocol that is easy to perform in a repeatable manner and mimics automated flying. This involves following similar flying patterns to automated flights whilst maintaining the flexibility of a manual flight; this way, the pilot maintains complete control of the drone and the area of interest, can rapidly respond to challenges such as bird encounters, and does not require an internet connection. To guarantee the required image overlap for orthomosaic reconstruction, and to ensure images were sharp and focused, the most critical parameters explored included the fixed horizontal flight speed, time interval of image shooting, shutter speed, aperture, and ISO. We hypothesised that both methods, when used appropriately, could obtain similar outcomes.

For automated flying, we tested a number of software options; these were: Map Pilot Pro from Maps Made Easy (free access with restrictions or a full license for $45/month; https://www.mapsmadeeasy.com*);* Drone Deploy (free trial option or a full license for $349/month; https://www.dronedeploy.com/); UgCS (full license for 59€/month; https://www.sphengineering.com/flight-planning/ugcs); and DJI Pilot (https://www.dji.com/de/downloads/djiapp/dji-pilot; DJI Pilot 2 version currently exists). The first two apps include image collection and web-based map processing. We evaluated the applicability of these programs by testing their use in over 22 colonies during the course of our study, considering aspects like whether an internet connection was needed, if mid-flight restart of the mission was possible, if camera focus could be controlled manually, whether the home point could be adjusted during the mission, and the possibility of performing multi-battery missions.

In both cases, we collected images in the JPG format rather than RAW, as the latter would require more storage capacity and longer processing times. Since our average flights collect around 350 images each, the storage and processing requirements for RAW images would be substantial. Ideally, image collection should occur during good light conditions. In the case of poor light conditions or where backlighting is an issue, changing to RAW may be preferable, since it allows for more powerful post-processing that can improve image quality before further use.

#### Videos versus still images

We focused on the use of still images over videos for several reasons. Firstly, still images are typically used by local wildlife managers, and we aimed to support their work by refining the techniques they are already familiar with. Secondly, we aimed to test the counting of marine iguanas using citizen scientists. These volunteers learn how to identify iguanas through a tutorial provided by us on the Zooniverse platform, which is designed primarily to analyse still images or videos. In still images — but not videos — a simple zoom option is available; this greatly facilitates analysis in our dataset where the iguanas are relatively small within the image due to the flight altitudes. Without the zoom option, videos require lower flying altitudes, which increases both the surveying time and risk of disturbance. Overall, counting iguanas from stills is simpler and thus requires less skill and training than would be the case if videos were used. Finally, our expert team found that counting iguanas from still image datasets was more streamlined and more repeatable than from videos. However, it should be noted that, due to time limitations, our comparisons here are far from exhaustive, and further work is needed to explore the viability of similar analysis using video rather than still images. However, in select cases — where rough sea conditions (a rare event since our monitoring season coincides with calm ocean conditions) and high cliffs presented a severe challenge to our usual flight approach — we monitored using video. When flying this way, we utilised the same procedure in terms of drone and camera positions as detailed above for the cliff and steep incline flights, but increased the horizontal flight speed (to 3 m/s), which allowed a site to be covered faster. From the videos, we then extracted single frames for later use. Since we rarely used video, our protocol described herein focuses on the process of collecting still images.

### Testing approaches for aerial image analyses

#### Software to reconstruct flat terrains

In marine iguana colonies on flat terrain, aerial images are collected in nadir mode (camera at -90°, i.e., facing straight downwards). From these, we create orthomosaics (detailed, geometrically correct and georeferenced 2D maps composed of several overlapping images) of the surveyed area. Here, we use drone photogrammetry — a process that involves the orientation, alignment, and identification of common features (tie points) across overlapping images collected from an object or surveyed area. Photogrammetry then entails the generation of a dense point cloud, which is created by triangulating the 3D positions of the identified shared points (modern photogrammetry workflows allow skipping this time-consuming step; however, it is recommended to perform it when reconstructing complex objects). Next, a 3D mesh (model) of the object or area — which connects these points to form a volume or surface — is created, and finally a texture, which uses the original image information (pixels), is added to the model (see Figure S1A). After this, an orthomosaic can be generated as a GeoTIFF file, corrected for size and geographic position by using the geographic coordinates embedded in the aerial images. Some software (e.g., Pix4Dmapper) uses a Digital Surface Model (DSM) to create an orthomosaic. A DSM is a three-dimensional representation of the terrain’s surface, including all its features, and it is created from the point cloud. The aerial images are then projected into the DSM, rather than being added as a texture.

Every photogrammetry software follows a similar process to generate the models, but each varies in terms of price, precision, ease of use, and processing capacity. Assessing the quality of photogrammetry products is vital for good results [32]. We evaluated four standard photogrammetry programmes: Agisoft Metashape v. 2.0 (https://www.agisoft.com/); Drone Deploy (https://www.dronedeploy.com/); Pix4Dmapper v. 4.9.0 (https://www.pix4d.com/); and Open Drone Map v. 3.5.0 (https://www.opendronemap.org/). Most are desktop-based and require powerful computers (e.g. at least 32 GB RAM, 8–23 GB VRAM), except for Drone Deploy, which is web-based and requires a good internet connection.

To assess the degree to which the quality of the orthomosaics produced could affect aerial counts, we employed an analysis that compared the number of marine iguanas identified in an original aerial image with the number of marine iguanas reconstructed in the corresponding orthomosaic. For this, four selected original images — each featuring iguanas — were projected onto the orthomosaics. We used the scale-invariant feature transform algorithm (SIFT; [34]) to extract the features (labelled iguanas) from the original image and matched these features to the orthomosaic by using the deep neural network LightGlue [35]. The workflow is implemented in Python on GitHub (https://github.com/cwinkelmann/image_template_search). Projected labels were corrected using the Computer Vision Annotation Tool (https://www.cvat.ai) and compared against the original labels. A percentage of similarity was calculated for each image and with each software used. We tested three cases: an image with five iguanas (low level density) at James Bay (locally known as Puerto Egas) on Santiago Island, with 25 iguanas (medium level density) at Montura on Floreana Island, and with 223 iguanas (high level density) at Cabo Douglas on Fernandina Island (see Table S1 for information on sites selected and Figure S2 for the locations). Additionally, we explored a special extra case, also at James Bay. At this site, we also performed traditional ground-based capture-mark-resight — which involves surveying in two sampling occasions — alongside the drone surveys. On the first occasion, we flew the drone ahead of the surveyors over the entire area, but on the second occasion, the drone and the surveyors overlapped towards the end of the survey. We recognised that the iguanas were moving due to the physical presence of surveyors; this situation is atypical for our dataset but can occur when multiple surveying approaches are used in parallel, as sometimes happens during the time-limited monitoring expeditions. In this case, an iguana captured on one sweep of the drone during the flight path might not be in the neighbouring overlapping image when the drone returns, affecting the reconstruction of that individual and leading potentially to other artefacts — such as blurring and smearing — in the images. We used this as an opportunity to explore the effect of this scenario on the resulting counts. All these flights were conducted at an altitude of 25 m.

#### Software to reconstruct cliffs

Since nadir images cannot be collected on cliffs, 2D maps cannot be reconstructed. Instead, we fly with the camera facing the cliff diagonally (camera angle: -40°). For these images, we tested reconstructing the colonies by both generating panoramas as stitched JPG images (see Figure S1B) and by creating 3D models. For panoramas, we used the open-access and user-friendly Image Composite Editor (https://www.microsoft.com/en-us/research/project/image-composite-editor/; Windows only), although paid alternatives such as Adobe Photoshop (https://photoshop.adobe.com/discover) can also be used. In parallel, we trialled generating 3D models through photogrammetry using the Drone Deploy platform, which follows the same steps used to create an orthomosaic, but instead exports the 3D model. Although Agisoft Metashape and Pix4Dmapper can also generate 3D models, we have typically used Drone Deploy for reasons of brevity and simplicity.

### Comparison of different counting methods in the aerial imagery

#### Software and protocols to count marine iguanas in flat terrains

**Fig. 3:**
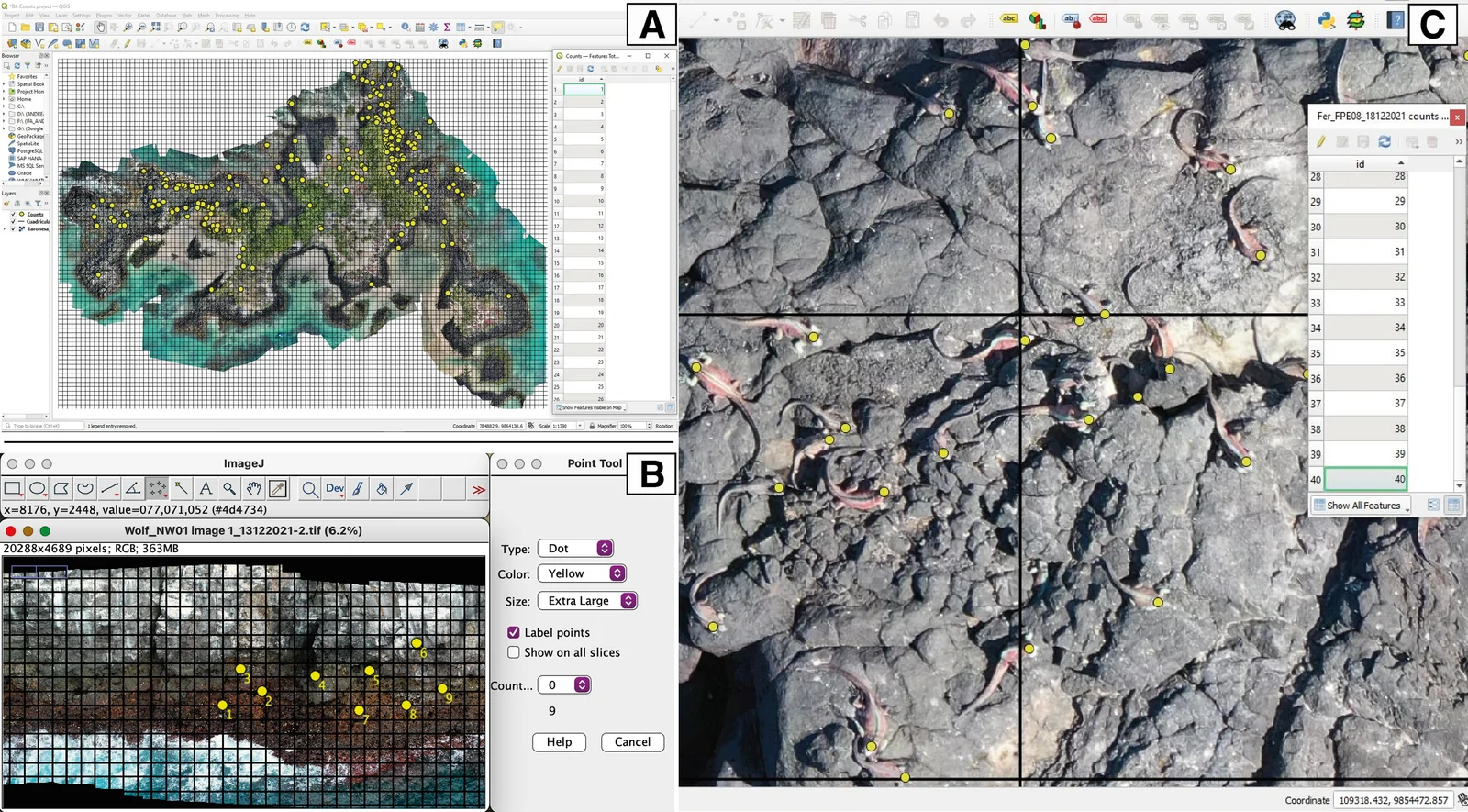
Our standardised process for counting marine iguana individuals in flat terrain using orthomosaics (**A**) and on cliffs using panorama images (**B**). In both cases, we create a grid and add points for each individual found. (**C**) A zoomed-in view of the iguanas found and marked in an orthomosaic

The final step of our aerial image analysis involves counting individuals in orthomosaics, panoramas, or 3D models. For counting in orthomosaics, we use the open-access geographic information software QGIS (https://www.qgis.org/), although commercial alternatives such as ArcGIS (https://www.esri.com/en-us/arcgis/geospatial-platform/overview) can also be used. The standard steps we developed for counting consist of inputting the GeoTIFF map, then generating a 4 × 4 m grid, and creating a new point layer to mark each individual found. The addition of this grid ensured the correct scale for the images, since without a frame of reference, it is easy to view the image at an inappropriate level. Next, we scan the orthomosaic on the computer screen following a horizontal zigzag pattern (moving across the image, then downwards and returning along that row in the opposite direction, with some overlap between each row, continuing until all the image has been viewed), adding a point on top of each iguana found (where possible, on the head), from which the geographic position could be derived (Fig. 3A). Tutorials on how to add geopoints to individuals in maps can be found online (e.g., https://maps.nls.uk/projects/urbhist/guides_vectorlayerqgis.html). Finally, we obtained the total number of iguanas by summing up all the geopoints. Highly trained team members conduct this counting. To assess how inter-observer bias could affect aerial counts, we compared individual counts among four team experts, who marked iguanas across 465 raw aerial images using the Hasty.ai tool (https://app.hasty.ai/). We calculated the coefficient of variation across the four total counts on images with and without iguanas (presence was defined as when three of four experts detected an iguana, and absence was defined similarly). Furthermore, we compared the total counts across three orthomosaics among three team experts and calculated the coefficient of variation among the outcomes.

#### Software and protocols to count marine iguanas on cliffs

For counting in panoramas, which lack geographic information, we used an image analysis software rather than a GIS program. Here, we used both ImageJ (open-access; https://imagej.net/) and Adobe Photoshop (paid; https://photoshop.adobe.com/discover). The standard process for counting involves inputting the JPG image and creating a grid (~ 1,000 × 1,000 pixels); ideally, one grid cell should fit the computer screen while keeping good resolution; see an example in Fig. 3B). The image was then scanned by eye using the same search pattern described above. In ImageJ, we used the multi-point tool, and in Photoshop, we created a new layer of points. The open-source software GIMP (https://www.gimp.org/) is a further functional option.

For counting in the 3D models, we used the Drone Deploy website platform. Here, we open the model with the 3D viewer, zoom in, and carefully follow the same standard search pattern, as a grid cannot be created. Each added point is then a georeferenced iguana.

### Traditional ground-based versus aerial counting

Direct counting of marine iguanas is typically conducted by GNP rangers on annual expeditions, during which more than 30 selected sites across the archipelago are visited and ground-based simple counts are performed. Essentially, this involves approaching the colonies closely with a small team of trained surveyors on foot who work together, moving through the colony and recording the total number of iguanas seen on that occasion. In November 2022, we joined one such 14-day expedition. During this work, we selected 10 sites to fly drones (see Table S1 for geographic information of the selected sites and Figure S2 for a map with locations). The sites included colonies with low, medium, and high abundances of marine iguanas and are situated on flat terrains as well as cliffs, thereby expanding the comparable dataset reported in our previous study, where we validated the aerial method in four colonies [29]. The surveying methods were performed in parallel. The GNP standard ground transects cover ~ 1 km on each side of a starting point at a colony. We flew the UAV over one side of the transect, flying 10–15 min before the ground surveyors began, to minimise the effect of potentially disturbing the animals during ground surveys. Most flights were conducted from a boat rather than from land (see Figure S3 for an illustration of the monitoring design protocol). To assess how counts varied across the methods used, we compared the counts generated from the ground with those obtained from the air (which had a non-normal distribution) using a paired Wilcoxon signed-rank test (*n* = 10 paired sites). Additionally, a paired t-test was conducted to compare the monitoring time (which had a normal distribution) required by each method. The software R was used for these tests.

## Results

Table S2 provides a summary of the relevant information we have identified regarding the remote sensing tools and skills required for collecting and analysing aerial imagery for the purpose of accurately counting animals.

### Standardised protocol for manually collecting high-quality aerial images

The automated flights produced high-quality orthomosaics from the beginning, since the necessary overlap between images was ensured. In contrast, our initial manual flights (field season 1) suffered from insufficient overlap, which impacted map reconstructions. However, in later field seasons, map reconstruction quality improved markedly once we found adequate methods for standardised manual flying. From this experience, we determined that the following settings produced good results for our chosen drone model: flying in tripod mode (fixed horizontal speed, H.S) of ~ 40/100, which represents 1.7–2 m/s, and using a 3-second image capture time interval with a shutter speed of 1/2,000; the rapid shutter speed prevents blurring of an image whilst the UAV moves. Motion blur (a visual distortion caused by movement during a long exposure time) values of less than one pixel represent a sharp, high-quality image [36]. Our recommended horizontal speed and shutter speed help reduce motion blur. Table S3 lists several combinations of horizontal speed and shutter speed that generate different motion blur values. For instance, we could fly at 3 m/s with a shutter speed of 1/1,000 and still capture sharp images; therefore, researchers should choose the combination of settings that best suits their purpose. Fixing the image ISO and aperture in a standard way is a challenge, since light varies constantly; therefore, these settings should remain automatic to adapt to varying light conditions, but can be set within an acceptable range. We recommend taking care to avoid capturing overexposed images, as they are more difficult to fix than underexposed images in post-processing. The use of an exposure warning option can be helpful here. In case of suboptimal light conditions, RAW photos could be taken since these allow a greater range of post-processing options, which may compensate for underexposure. We also found that using a rule of thirds — where the grid shown on the live image-feed from the UAV is used — can ensure the correct flight pattern. Here, we recommend using a ground object as a visual reference — moving the drone such that the object moves across the screen by around 33% in the horizontal part of the mapping pattern, and by 25% when moving forwards or backwards, between successive images. This, together with the 3-second image capture interval, enables a good overlap (> 75% to the front and 80% to the side when flying at a height of 25 m). Note that the overlap achieved using these settings will depend on the flight altitude and the size of the image collected by the drone. More details on overlap percentages resulting from various combinations of flight altitude, speed, and image capture time intervals are provided in Tables S4 and S5. An example of how the remote controller screen looks in an automated versus manual flight is shown in Figure S4; the settings shown here are from the Mavic 2 Pro; however, other models have similar options. When flying on cliffs, where automated flights are not possible, we utilise the same settings but manoeuvre the drone in front of the cliff whilst moving along it, maintaining a steady parallel distance. Here, the grid is not relevant since we don’t follow the same flying pattern as used on flat terrains. Flying faster — e.g. at 3 m/s — could be possible since a good side overlap can be achieved (Table S5).

We recommend always using a co-pilot to help ensure safe flying and avoid damage or loss of the drone resulting from obstacles; in our experience, the obstacle avoidance system can fail, especially in low-light conditions, such as shadows created by tall rock faces. Table 2 summarises the relevant parameters for conducting standard manual flights and ensuring safe operations when flying from land or a boat.

**Table 2:** Recommended parameters for efficient and safe automated and standardised manual flights from land or boat, based on the DJI Mavic 2 Pro drone. Please note that other models from this manufacturer typically offer similar options

Flight Stage	Parameter	Land Setting	Boat Setting	Justification
Pre-flight Remote Controller (RC) setup	Map display	Activated	Activated	Helps locate the drone and facilitates a rapid return
	Custom RC1 Button	Camera facing down/forward	Camera facing down/forward	Allows rapid change of camera angle. Secures nadir position
	Custom RC2 Button	Obstacle avoidance	Obstacle avoidance	Avoids rapid enabling/disabling of the obstacle avoidance system, helps when landing in the boat and catching by hand
	Maximum drone-RC distance	1 km or less	1 km or less	Reduces the chances of signal loss due to distance and helps to recover VLOS faster
	Downward vision positioning	Enabled	Disabled for cliffs	Enable: ground-based recognition prevents drift. Disable: Moving water can cause the drone to drift
Pre-flight Camera setup	Shutter speed	1/2,000	1/2,000	Fast shots avoid motion blur
	Continuous autofocus	Enabled	Enabled	Avoids unfocused images
	ISO & Aperture	Auto	Auto	Adapts to changing light conditions, reducing the need for post-processing of images
	Exposure warning	Auto	Auto	Indicates the need for adjustment of the exposure value depending on the light conditions
	White balance	Auto	Auto	Adapts to changing light conditions
	Image format	JPG	JPG	Saves storage space and is captured quicker than a raw image
	Grid	Enabled	Enabled	Allows the pilot to use the rule of thirds to maintain the correct flight path, allowing necessary side overlap (> 75%)
	Image interval shooting	3 s	3 s	Allows necessary frontal overlap (> 75%). Resembles the time interval used in automated flights
	Video captions	Enabled	Enabled	When taking videos, it generates a SubRip Subtitle (SRT) file with geospatial coordinates of the frames
In-flight position and movement	Flight altitude	Flat: 20–30 m; Cliff: 15 m	Flat: 20–30 m; Cliff: 15 m	Allows suitable image collection depending on the average local body size (see Methods) of the target and the terrain surveyed
	Flight pattern	Flat: Start perpendicular to the coastline and fly in a forward-side-backwards pattern Cliff: Start perpendicular to the cliff and fly parallel to the rock	Flat: Start perpendicular to the coastline and fly in a forward-side-backwards pattern Cliff: Start perpendicular to the cliff and fly parallel to the rock	Allows the necessary overlap for image collection and area reconstruction
	Tripod mode	Horizontal speed: 30–40/100 (1.7–2.0 m/s); Vertical speed: 100/100	Horizontal speed: 30–40/100 (1.7–2.0 m/s); Vertical speed: 100/100	Constant horizontal speed during the flight allows for the necessary overlap. Resembles the speed used in automated flights Flexible vertical speed allows for rapid response (e.g. to avoid collision with birds)
	Visual line of sight (VLOS)	Maintained	Maintained	The drone should be visible at all times; recommend using a co-pilot and maintaining an appropriate distance from the boat to the shore. Binoculars can help
In-flight adjustments	Obstacle avoidance	Enabled throughout	Enabled after launch and disabled before landing	Enable: Avoids collisions Disable: Necessary when catching drones by hand or landing on a boat
	Gimbal angle	Flat: Nadir (-90°) Cliff: Diagonal (-40°)	Flat: Nadir (-90°) Cliff: Diagonal (-40°)	Collects images at the most suitable angle for the terrain
Return to Home (RTH) and safety measures	Connection lost	RTH	RTH or Hover	RTH: Allows the drone to fly back towards the pilot automatically. When used from a moving boat, the home point, in this case, the remote controller, must be updated constantly Hover: Allows the pilot in a moving boat to approach the hovering drone and recover the signal. Not recommended on land
	RTH altitude	100 m	100 m	Allows ascending to recover the signal if lost and avoids collision with objects like trees and cliffs when returning
	Smart RTH	Enabled	Enabled	Allows the drone to calculate the time needed to return safely to its home point
	RTH obstacle avoidance	Enabled	Enabled	Avoids obstacles when returning
	Home point update	Constant	Constant	Updates home point constantly if flying from a moving boat or walking on land, facilitating the return of the drone
Landing	Landing protection	Enabled	Disabled	Enable: Helps the drone when landing by verifying the terrain Disable: If landing protection is enabled, the drone will avoid colliding with the person attempting to catch it from the boat

### Relevant parameters for flying autonomously

When choosing automated flights, either for repeatability or ease of use, several software options are available. During our monitoring in the Galápagos, we successfully tested several applications for automated flying. Table 3 provides a summary of the most significant advantages and disadvantages of each, which we identified as relevant during our surveys at remote and/or otherwise challenging sites. Overall, we find DJI Pilot to be the most cost-effective option, followed by UgCS. Maps and digital surface models (DSMs) for terrain awareness can be downloaded/imported to be used offline in mission planning. Drone Deploy and Map Pilot Pro remain internet-dependent for mission planning. DJI Pilot 2 is the newest version of the DJI native app, and despite not being tested, it has been added to the table for comparison.

**Table 3:** Comparison of key parameters for aerial monitoring among five software programs that facilitate automated drone flights

Parameter/Software	Map Pilot Pro	Drone Deploy	DJI Pilot	UgCS	DJI Pilot 2
License to use	Paid $15/month	Paid $349/month	Free (Included in SRC*)	Paid $59/month	Free (Included in SRC*)
Supported Operation system	iOS, Android**	iOS, Android	NA	iOS, Android	NA
Offline mission planning (previously downloaded maps required)	✗	✔***	✔	✔	✔
Multi-Battery Mission option (e.g. pause to change battery and resume)	✔	✔	✔	✔	✔
Offline terrain awareness (require digital surface models previously imported)	✗	✗	✔	✔	✔
Obstacle avoidance during the whole flight	✔	✔	✔	✔	✔
Movable Home Point (allows update of home point before landing)	✔	✔	✔	✔	✔
In-Air start and restart (start mission in-flight and resume mission if it was paused mid-flight)	✔	✔	✔	✔	✔
Manually focus the camera during the mission	✔	✗	✔	✔	✔
RAW format for the images collected during the mission	✔	✗	✔	✔	✔
Video option during the mission	✔	✔	✗	✗	✗
Import KML files (polygons) as missions	✔	✔	✔	✔	✔
Live Map (orthomosaic reconstruction preview during the flight)	✗	✔	✗	✔	✗
Image post-processing option included in the app	✔	✔	✗	✗	✗
Type of drones supported	Consumer	Consumer	Consumer	Consumer	Commercial/ Professional (e.g. Enterprise series)

*DJI Smart Remote Controller; SRC; **Limited support; ***Up to 10 routes created online but modifiable offline

### Outcomes from image processing software

Based on our defined flight altitude (25 m), the resultant GSD values — as determined by the software — were 0.93 cm/pixel in Drone Deploy, 0.63 cm/pixel in Agisoft Metashape, and 0.60 cm/pixel in Open Drone Map and Pix4Dmapper for all sites. For UAV photogrammetry, these GSD values indicate a high resolution and spatial accuracy. Regarding the success of each software for reconstructing marine iguanas in orthomosaics, on images with low to medium density of individuals, the commercial software generally obtained higher percentages of reconstructed iguanas (Table 4) than the open-access alternative (Open Drone Map). This means that the iguanas identified in the orthomosaics are all or nearly all of those captured by the drone during the survey, and that few or none were ‘lost’ to reconstruction artefacts (unwanted visual structures not present in the original surface, for example, motion blur). See an example of reconstructed iguanas identified in an orthomosaic in Figure S5.

**Table 4:** Comparison of the number of marine iguanas reconstructed in orthomosaics using four photogrammetry programs. The values in parentheses represent the percentage of iguanas reconstructed by each software compared to the number of iguanas present in the original image of each site. The sites represent surveys at four colonies, with varying abundances, in the Galápagos. The colony marked with an asterisk (*) indicates an aerial survey where the drone overlapped with ground-based surveyors, resulting in disturbance and movement of the iguanas during the flights — educational license option (**)

Site (Island)	Iguanas counted in the original image	Iguanas counted in the reconstructed area (%)			
		Agisoft Metashape	Drone Deploy	Pix4Dmapper	Open Drone Map
Montura (Floreana)	5	5 (100%)	5 (100%)	3 (60%)	0 (0%)
James Bay 1 (Santiago)	25	25 (100%)	25 (100%)	25 (100%)	20 (80%)
Cabo Douglas (Fernandina)	223	197 (88%)	210 (94%)	215 (96%)	199 (89%)
James Bay 2* (Santiago)	9	7 (78%)	4 (44%)	7 (78%)	7 (78%)
**Price**		$3499/$549**	$349/m	$241/m	Free

It seems that a high density of iguanas may influence the reconstructions, with the dense colony at Cabo Douglas — where more than 200 individuals can be captured in a single image — being a notable example. Here, all software tested failed to reconstruct all the iguanas, with Agisoft Metashape being the worst and yielding an outcome comparable to that of the free Open Drone Map (Table 4). In crowded colonies, iguanas tend to constantly react to close individuals by repositioning, which could affect the success of reconstruction. These movements generate blurry objects; however, individuals can still be partly recognisable.

Interestingly, results from the fourth case analysed — where the iguanas were disturbed by surveyors on the ground and relocated themselves during surveys — could indicate that significant movement influences the reconstruction of individuals. In this case, Drone Deploy obtained the lowest reconstruction percentage, which could mean that this software is stricter in the sense that if an object is not present in all overlapping images, it does not reconstruct it, not even as a blurred object. It is worth noting, though, that the dataset used in this test is small and may not be representative of all cases. Nevertheless, these results do provide some insights into software differences in reconstruction success.

Because cliffs are not reconstructed as orthomosaics, but as panoramas or 3D models, this analysis could not be performed. However, these should achieve reconstruction success similar to orthomosaics because the images are collected under the same parameters, and the colonies behave in a similar way. It is worth noting that creating panoramas requires more time, as it involves manual processing, whereas generating 3D models is typically done directly on a website platform or via batch processing in the software. Nevertheless, counting in panoramas on ImageJ or Photoshop is easier thanks to the use of a grid, whereas 3D models necessitate more careful movement during scanning by eye, using a vertical zigzag pattern, which a trained person should perform.

### Traditional ground-based surveys versus aerial counts

In tests of inter-observer variability of counts across a dataset of 465 aerial images, the average overall agreement among the experts was 96%, while the average agreement considering only images with iguanas present was 83%. In comparisons of total expert counts across three orthomosaics, the average agreement was above 90% (see Tables S6 and S7 for further details and raw data).

Our analysis comparing ground-based counts versus aerial-based counts reaffirms that drone surveys yield higher counts (average 1,299 ± 2,456 iguanas) than ground surveys (average 1,232 ± 2,410 iguanas; see Table 5) across all terrains and abundances (e.g. on the cliff of Champion Islet and in the very crowded colony of Cabo Douglas). The paired Wilcoxon signed-rank test showed that this difference was significant (V = 55, *p* = 0.0059). Moreover, aerial surveys take significantly less time (average 46 ± 12 min) to collect data than ground surveys (average 82 ± 37 min; *t* = -2.95, df = 9, *p*-value = 0.01623; Figure S6). However, counting individuals in the orthomosaic is laborious, especially in densely populated colonies, which are also naturally the sites that require more time to survey using traditional ground-based methods.

**Table 5:** Comparative results of marine iguana counts conducted by aerial and ground monitoring performed in parallel. The abundance is based on counts from drone imagery: low < 150 individuals, medium 150–500, high >1000

Site (Island)	Abundance	Marine iguanas counted		Surveying time (mins)		Distance surveyed (Kms)
		Ground counts	Drone counts	By foot	By drone	
Cabo Marshall (Isabela)	Low	0	8	96	41	1.1
Seymour North (Santa Cruz)	Low	24	43	117	40	1.1
Champion Islet (Floreana)	Low	35	50	39	43	1.6
Isla Lobos (San Cristóbal)	Low	45	87	60	63	1.8
Cabo Ibetson (Pinta)	Medium	143	218	69	38	1.1
Baronesa Islet (Floreana)	Medium	150	270	32	33	1.4
Mosquera (Santa Cruz)	Medium	152	193	95	67	1.6
Gardner Islet (Española)	Medium	371	430	63	55	2.7
Punta Mangle (Fernandina)	High	4655	4733	98	46	0.9
Cabo Douglas (Fernandina)	High	6753	6958	154	43	1.2

## Discussion

The trade-offs and decisions researchers make when implementing UAVs in their studies (e.g., flying higher to cover a larger area but losing image resolution, or choosing cheaper photogrammetry software but compromising product quality) will affect the overall level of detection error [37] — fortunately, standardised protocols help reduce it. This work highlights the development of a protocol for the efficient and safe collection of high-quality aerial images from land or boat across coastal areas of the Galápagos archipelago — simulating automated flights whilst maintaining the flexibility of manual flying — yet collecting good quality data to generate accurate reconstructions. This is a good solution when advanced but more expensive equipment for automated flying cannot be used. Moreover, the standardised steps for image analyses documented herein allow for the optimisation of a colony reconstruction as orthomosaics, panoramas, or 3D models, thus aiding further accurate object counting by experts, citizen scientists, or potentially artificial intelligence. Applying these protocols in future continuous monitoring campaigns will help to accurately monitor the status of the populations, thereby strengthening their conservation management.

### Insights on the development of the aerial monitoring method

#### Quality of the data

The quality of the collected data is of utmost importance for reliable data analysis, interpretation, and further use [37]. Our analysed orthomosaics have a Ground Sampling Distance (GSD) of 0.6–0.93 cm/pixel, which, based on our object size (SVL: 12–49 cm; [21]), indicates that our adult individuals are represented by 21 pixels or more (object size divided by the GSD value). Ideally, the object to be examined should be represented by at least three pixels in the image [24]. In our case, considering the similarity of the background to the object, and the aggregation patterns often exhibited by the iguanas, we determined that the images’ GSD values obtained (0.46–0.69 cm/pixel) by flying at an altitude of 20 to 30 m (depending on the SVL of the subspecies) are needed to obtain high-quality data, at least with the Mavic 2 Pro. Flying at a higher altitude will increase survey speed but simultaneously increase GSD and reduce resolution; the best compromise between these will be case-specific, and protocols should be adapted for each purpose.

#### Pilot-controlled flying

Although conducting automated flights ensures the collection of high-quality data and repeatability over time [38, 39] — which is recommended for continuous monitoring — this is not always the best option in the field. Automated flights need planned sampling areas, which is not realistic for expeditions where work must be performed without prior knowledge of the colonies, or internet access, which is required for creating the polygons to be surveyed when using low-budget equipment. Although advances — such as the expanding availability of the internet (e.g., via Starlink; https://www.starlink.com/) and other satellite-based providers — are enhancing global connectivity, internet access may still pose a critical limitation in extremely remote locations. These services can be influenced by weather patterns, regional restrictions, and satellite coverage, and may be expensive in the context of longer field seasons. Software developments mean that it is now possible to download maps and digital surface models to plan offline missions in native DJI applications, such as DJI Pilot and the advanced DJI Pilot 2. However, it is worth mentioning that the latter option requires more expensive hardware.

Moreover, automated take-off usually necessitates stable conditions; in our case, attempting to fly primarily from a boat has always made it preferable to have a pilot-controlled take-off. If the software used to fly autonomously has the option to upload and start the mission in-air, an automated flight can be undertaken after a manual take-off. Additionally, an efficient flight will focus solely on areas of interest. Since the target area of coastline is tide-dependent, defined polygons should always be executed under the same tide conditions, ideally low tide for this species, to prevent unnecessary flights over water. Where tidal stage is important for deciding the extent of the area to be surveyed, survey timing would ideally be planned to match the optimal tidal level, in which case the survey area is predefined and automated flights can be used. However, remote regions often necessitate logistically complex and expensive expedition-based surveying, where sites must be visited in a time-efficient manner, thus precluding such adaptations. In this case, manual flight can allow for more dynamic surveys that readily adapt to the conditions presented.

We also found that the overlap we obtain (76% to the front and 84% to the side), with the defined settings when manually flying, falls within the recommended range (70–80%, [33]). A heterogeneous terrain, such as the Galápagos coastlines, facilitates the identification of common points between the overlapping images. Monitoring a more homogeneous terrain — such as a tropical rainforest — may require greater overlap in the images (80–85% to the front and side). For a pilot who already has the skill to fly a drone, learning the standardised manual flight method requires little training (perhaps 2–3 days). For a complete novice, more extensive training may take at least a week, with experienced support needed during the initial phase of flying.

#### Still aerial images

Collecting still aerial images in our case works well, as our subjects move very little — this is shown in the successful reconstruction of iguanas in the orthomosaics. However, images require substantial time for collection to ensure enough overlap. In contrast, videos can cover an area faster than overlapping still images. Their frames can be extracted to reconstruct the area, but in doing so, we may lose resolution, which is essential to identify iguanas correctly. A further consideration is image resolution — in the case of the Mavic 2 Pro drone, still images have a higher resolution (20 megapixels; 5,472 × 3,648 pixels) as compared to video (8.3 megapixels; 3,840 × 2,160 pixels). Furthermore, collecting data via still images may require less storage capacity. To date, videos collected via UAV have primarily investigated the abundance and behaviour of larger and more mobile species [40]; we are unaware of any published work that explicitly aims to compare both methods directly. In a study that tested automated detection of cetaceans in drone footage, researchers found that still images performed better than video-based data for individual detection [41]. At present, drone-based surveying is more commonly performed using still, rather than video, imagery [42]. Creating a specific platform for analysing videos (e.g., with the ability to zoom) or using artificial intelligence-based approaches for analysis could enhance the applicability of video surveys for monitoring smaller, sedentary species such as marine iguanas. Using videos could also expand the population-monitoring study to investigate behaviours.

#### Site reconstruction

In our case, creating orthomosaics has proven to be the most effective option for reconstructing colonies in flat terrain. From our small test, we identified that the software Pix4Dmapper obtained the highest reconstruction rates of iguanas in orthomosaics. Previous work that compared photogrammetry software has shown minor variability in reconstruction. An underwater study found that Agisoft Metashape has a better capability of aligning images at larger spatial scales, while Pix4Dmapper shows a higher spatial accuracy and lower reprojection error [43]. Since animal movement seemed to play a role in reconstruction success, further investigation should be performed on detection errors relating to this. There are two main types: omission, where an individual is not reconstructed because, while moving, it was not captured by all overlapping images, or duplicates, which occur when a moving animal is captured in a different location on overlapping images and is thus reconstructed more than once [44].

Several colonies of marine iguanas are located on cliffs — meaning that other reconstruction methods, such as panoramas or 3D models, are needed. Panoramas are suitable; however, these lose the image’s geographic information. Furthermore, since the profile of an island is curved, the entire set of images cannot be used at once to create the panorama; therefore, one flight needs to be reconstructed in sections, which requires more time. Finally, since a panorama is a flat image, it may lack specific parts of the cliff (e.g. behind a rock), which a well-done 3D model can capture while maintaining the geographic location [24]. Georeferenced data — as derived from 3D models and orthomosaics — has the advantage that it can be further used to explore spatial questions. For instance, to calculate the length and area covered by aerial surveys, calculate metrics on colony density and distribution patterns, or to measure the size of individual marine iguanas. Nevertheless, counting individuals in a panorama is a more straightforward process.

#### Counting

Our results reconfirm that aerial surveys consistently provide higher counts of individuals than ground-based surveys; these higher figures have been shown to more closely match estimates from the capture–mark–resight approach [29], and thus are unlikely to represent an overestimation of the population size. Moreover, we further extend this previous work to include a broader type of terrain and a larger range of abundances — meaning that this method can be confidently used in any colony, including cliffs, if guidelines are followed. Implementing drones in future monitoring campaigns will reduce the surveying time of a site, allowing surveyors to either complete expeditions faster, use the remaining time to explore new sites, or start processing images in the field. In terms of inter-observer reliability — when considering the overall dataset or the total counts in the orthomosaics — the difference between counts was always below 10%. When considering only images featuring iguanas (i.e., removing ‘empty’ images), the difference was higher, at 17%. However, it is important to mention that in our dataset, the majority of images with iguanas feature a low number of iguanas (five or fewer), meaning that each missed iguana adds significantly to the calculated discrepancy, even though the actual number of ‘missed’ iguanas is relatively few (Table S6). As with any surveying effort, minor inter-observer differences in counts are to be expected, and with traditional ground-based counts, it is often not even calculated. Moreover, since images and information collected from aerial surveys can be readily reanalysed, further evaluations for a range of purposes are possible; notable uses could be aiding research on demographics, movements, breeding aggregations, behaviours and habitat preferences. Advances in automated analysis — such as those enabled by developments in artificial intelligence — could render such datasets increasingly valuable, as confirmed by other work [45].

#### Best practices

Based on the work carried out in the James Bay colony of Santiago Island — where the aerial surveys were conducted at the same time as the ground-based surveys, causing disturbance and movement of the marine iguanas — we see that the individuals there were not accurately reconstructed in the orthomosaics. This is in contrast with the aerial-only surveys. This finding supports our earlier observation that drones — when flown carefully and at a reasonable altitude — disturb less than ground-based surveys. This may contribute to the consistently higher detection in aerial surveys when compared to land-based counts, confirming the findings of several other investigations that have demonstrated the low level of disturbance caused by drone surveys on wild populations [46–49]. The impact of drones on wildlife remains a topic of ongoing discussion. Some studies have shown that factors reducing individuals’ reactions include take-off distance, flight angle and speed of approach, and the flight design — specifically whether it has a fixed pattern or not [50–52]. In our case, launching the drone on land from a distance of approximately 30 m from the individuals — and even further from a boat approaching the colony — at a minimum predefined height, and maintaining a flight speed of ~ 2 m/s, has helped reduce disturbance. During our expeditions, we have witnessed this in marine and land iguanas, as well as in pinnipeds and sea turtles. Colonies of breeding birds, though, should always be considered more cautiously [46]. When our drones were approached by birds, such as frigate birds (*Fregata magnificens* & *F. minor*) or Galápagos hawks (*Buteo galapagoensis*), manoeuvring the drone upwards and backwards, away from the bird, has demonstrated effectiveness [24] and highlights another advantage of controlling the flight through the standardised manual flying protocol.

### Limitations of the method and recommendations

Importantly, the rapid obsolescence of technology necessitates adapting protocols to new alternatives. In our case, we developed our protocol based on the DJI Mavic 2 Pro (GSD = 0.46–0.69 cm/pixel at 20–30 m height), which at the time of our study was ideal, due to its camera features, size, flight time, and price. This drone is now discontinued, and some newer models may provide more significant advantages, but at a higher cost (Fig. 2). The following current model is the Mavic 4 Pro (https://www.dji.com/de/mavic-4-pro), which is significantly more expensive (~ 2,700 €) but has a GSD of 0.10–0.15 cm/pixel at the survey altitudes. Other similar alternatives are the Mavic 3 Pro (https://www.dji.com/de/mavic-3-pro), which is more expensive (~ 1,900 €); however, it features a 4/3” CMOS Hasselblad camera with 20 MP resolution and a zoom option, weighs 958 g, has a longer flight time than the Mavic 2 Pro (up to 43 minutes) and has a GSD of 0.27–0.41 cm/pixel at the survey altitudes. One further option is the Mavic 3 Enterprise (https://enterprise.dji.com/de/mavic-3-enterprise), which has similar specifications, a higher price (3,500 €), a GSD of 0.53–0.80 cm/pixel at the survey altitudes, but includes an RTK (Real-Time Kinematic) system and a thermal camera. Also noteworthy are the DJI Air 3S and DJI Mini 4 Pro, which feature similar cameras, are lighter and less costly and also have a better GSD than the model we used. All these models (except for the Mavic 3 Enterprise) need a third-party app to create area-mapping routes used for automated flying (e.g. Drone Deploy or Litchi) or the native app DJI Pilot. The Enterprise series can support DJI Pilot 2 (advanced option) for this task. Both native apps support the use of downloaded maps for offline mission planning. DJI Phantom drones have also been widely used in wildlife monitoring studies [53], as they offered pragmatic features at lower prices. However, they have also been discontinued, and their larger size poses logistical challenges in remote locations. Although discontinued drones remain useful, buying parts and batteries can become a problem; this is a consideration when choosing whether to continue using a certain model or adapt to evolving technology. Multispectral drones could be used for detecting iguanas if a consistent difference in reflectance between the iguanas and the surrounding rocks or sand is found. In this sense, it would be essential to assess the use of such technologies across a variety of conditions, such as time of day, air temperature, solar intensity and thermal behaviour of the iguana.

Secondly, the capacity for data analysis must be considered. Drones collect more precise data than do ground-based methodologies [54] and allow the possibility for future use of those datasets, since they can constitute a visual time-capsule of the area surveyed and facilitate reanalysis. However, the large amounts of information involved can be laborious to process. In our case, an online citizen science (CS) method has helped us expedite the analysis. However, implementing our work via CS, in addition to its integration and validation, still required significant preparation time, constant communication with volunteers, and active promotion. Further avenues that leverage rapid developments in artificial intelligence (AI) are highly promising [45] but were not used here because our initial pilot work demonstrated that an AI-based approach would need significant development, given the highly variable and complex dataset we are working with.

Additionally, monitoring sites on cliffs remains a large challenge when using drones [55]. For the marine iguana, this is especially true when surveying tall cliffs, such as those found at Wolf Island, or on the south coasts of Floreana and Española islands. During surveys in such locations, the drone is easily camouflaged by the background of the cliff, making it hard, if not impossible, to follow visually, and it can easily lose signal due to the blocking effect of the rock. Moreover, the complex environment and shadows created can render the obstacle sensors incapable of accurately sensing distance, which can easily lead to a collision and loss of the drone. Therefore, extra care is needed if the target terrain is a cliff; for this, we outline specific safety measures (see Table 2).

Nevertheless, despite these challenges, drones enable the rapid surveillance of large areas, gather precise data quickly that can be archived and reanalysed for future questions, and provide access to remote locations while safeguarding pristine areas and circumventing dangerous boat landings. It is worth noting that since drones can capture multiple targets simultaneously, no traditional method is comparable. Monitoring multiple species or research questions during such flights can enhance collaboration, saving time, money and effort, whilst sharing the analysis workload.

Finally, based on our experience, we offer some recommendations to take into account before, during, and after the use of this methodology:

1. Verify whether the area where the drone will be used requires the pilot to have a license and/or permission to fly, since in several countries, such as Ecuador, flying drones weighing more than 250 g for non-recreational purposes requires certification, drone registration, insurance, and authorisation [56];
2. Obtain up-to-date information regarding nearby geo-fenced areas (e.g. when close to airports) and local flying regulations, to guarantee uninterrupted flying;
3. Communicate flight plans to local communities and authorities when applicable, to prevent potential conflicts;
4. Use a co-pilot — who maintains uninterrupted visual contact with the drone — to alert of approaching aggressive birds, help perform safe flights around cliffs and aid with take-off and landing;
5. If possible, obtain prior information about the terrain to be surveyed, the locations and boundaries of the colonies and daily/annual behavioural patterns, in order to capture the population when most individuals are present and visible;
6. Maintain flight logs (i.e., record data on time, flight altitude, observations on weather, tide, etc.) to allow effective evaluation of settings and problem-solving where needed;
7. Outline and practice clear emergency and contingency plans that are suitable for the common challenges of the local environment;
8. Perform regular equipment maintenance, especially after longer bouts of fieldwork, and/or where marine environments are concerned, and undertake regular visual inspections of fragile parts of the drones, such as the legs and propellers. Protocols to maintain a functional drone may be provided by the manufacturer, and work can be performed at certified maintenance centres;
9. Label and organise collected images and orthomosaics systematically. Ensure that the coordinates obtained from all images are accurately geographically located on the map. If multiple flights are performed manually in an area, avoid overlapping them significantly. To do this, in DJI drones, a path can be recorded in the remote controller. Otherwise, before reconstructing the flights, enter the image coordinates on a map. If there is overlap, either reconstruct a combined mosaic of all those images or delete the overlapping images. This is a crucial step to avoid duplicate counts of the same object on adjacent mosaics;
10. Follow a standard marking system when labelling objects of interest in the images. For example, in our case, marking all the iguanas on the head could facilitate later work, such as training machine learning algorithms or using clustering algorithms to aggregate citizen science data.

Our protocols relate to monitoring in the Galápagos Islands; however, they can be adjusted on a case-by-case basis, choosing a suitable altitude, optimal GSD (depending on the object size), preferred flight methods (automated or manual, from land or boat), image overlap needs, approach for mosaic generation, and analysis needs. While this article concentrates on marine iguanas, it offers guidelines for employing drones in remote sensing that can be tailored to various targets, such as other species in the archipelago like sea lions (*Zalophus wollebaeki*) and fur seals (*Arctocephalus galapagoensis*), various sea turtles and bird species, vegetation and macroalgae coverage, manmade objects such as plastics and wood debris as well as surveillance of the environment and human activities such as (illegal) fishing. With the rapid pace of technological advancements in both data collection and analysis, drone-based aerial surveys are becoming an increasingly valuable tool in conservation biology, enabling broader and more versatile applications across a range of species and environments. To support the adoption of drones in biodiversity monitoring, it is essential to inform local managers about the benefits of their use, such as improved access to wild populations, enhanced survey efficiency and collection of reusable high-resolution data, while also addressing key legal, logistical, and ethical considerations. Providing clear guidelines, hands-on training, and concrete examples of successful applications — such as that presented here — are key ways in which scientific researchers can offer practical support and help ensure the effective implementation of novel technologies in conservation.

## Supplementary Information

Below is the link to the electronic supplementary material.

Supplementary Material 1: Additional file 1: Lessons learned from surveying Galápagos marine iguanas using drones. Supplementary figures of the described protocol for image collection and analyses.

Supplementary Material 2: Additional file 2: Lessons learned from surveying Galápagos marine iguanas using drones. Supplementary tables of the described protocol for image collection and analyses.

## Data Availability

All data analysed in this study are included in this article or as supplementary information.
