# Supplementary material for: Lessons learned from surveying Galápagos marine iguanas using drones
Source: Front Zool. 2026 Jul 4;23:35. doi: 10.1186/s12983-026-00623-4 (PMC13621696; doi:10.1186/s12983-026-00623-4)
Supplement: Supplementary file 1 [file 12983_2026_623_MOESM1_ESM.docx]

**Figure S1. Aerial image post-processing.** A visual representation of the photogrammetry method for reconstructing flat terrains is shown, with the overlapping raw images aligned with the reconstructed area (orthomosaic) below (A). Additionally, a visual representation of the raw overlapping images selected before creating a panorama (composite image) is displayed in the software (B).

**Figure S2. Map of the Galápagos Islands, Ecuador, South America.** The marks shown on the map are the selected sites surveyed during the “emblematic species” expedition of the Galápagos National Park (GNP) service during November 2022. Further details of the sites are found in Table S1.

**Figure S3. Design of a system for monitoring a colony, using drones and ground-based surveyors, in parallel.** The Galápagos National Park (GNP) protocol on land consists of two transects, each 1 km in length, to either side of a starting point. When the drone is incorporated, it should monitor before the surveyors along the entire transect.

**Figure S4.** **Visualisation of the screen in the remote controller of a drone during an automated flight (above) versus a standardised manual flight (below).** The arrows indicate the parameters used by both methods to collect overlapping aerial images.

**Figure S5. Representation of the template search process.** An original image, which will have labels (points) for all the iguanas, is projected in the orthomosaic, then the labels glued on the orthomosaic are adjusted to match the reconstructed iguanas. All the points without an iguana near are removed, meaning those iguanas were not reconstructed.

**Figure S6. Boxplot comparing the time needed to cover an area using drones and ground surveyors on ten sites surveyed in the Galápagos.** The line in the middle of each box represents the mean for that method.
