# Supplementary material for: Lessons learned from surveying Galápagos marine iguanas using drones
Source: Front Zool. 2026 Jul 4;23:35. doi: 10.1186/s12983-026-00623-4 (PMC13621696; doi:10.1186/s12983-026-00623-4)
Supplement: Supplementary file 2 [file 12983_2026_623_MOESM2_ESM.docx]

# Table S1. Geographic Information of the sites used in this study. It includes the island, geographic coordinates and the analysis performed on each.

| Island | Site | Latitude | Longitude | Analysis done |
| --- | --- | --- | --- | --- |
| Española | Gardner Islet | 1° 20.589'S | 89° 38.665'O | Ground versus aerial surveys |
| Fernandina | Cabo Douglas | 0° 18.254'S | 91° 39.190'O | Ground versus aerial surveys  Template search |
| Fernandina | Punta Mangle | 0° 27.370'S | 91° 23.343'O | Ground versus aerial surveys |
| Floreana | Baronesa Islet | 1° 13.649'S | 90° 26.658'O | Ground versus aerial surveys |
| Floreana | Champion Islet | 1° 14.319'S | 90° 23.092'O | Ground versus aerial surveys |
| Floreana | Montura | 1° 18.791'S | 90° 30.459'O | Template search |
| Isabela | Cabo Marshall | 0° 1.127'S | 91° 12.445'O | Ground versus aerial surveys |
| Pinta | Cabo Ibetson | 0° 32.666'N | 90° 44.252'O | Ground versus aerial surveys |
| San Cristóbal | Isla Lobos | 0° 51.354'S | 89° 33.948'O | Ground versus aerial surveys |
| Santa Cruz | Mosquera | 0° 24.296'S | 90° 16.703'O | Ground versus aerial surveys |
| Santa Cruz | Seymour North | 0° 24.041'S | 90° 17.459'O | Ground versus aerial surveys |
| Santiago | James Bay | 0° 14.510'S | 90° 51.776'O | Template search |

**Table S2**. **Summary of remote sensing tools and skills required to collect and analyse aerial imagery to obtain a count of individuals or objects.**

| **Category** | **Tool/Software** | **Skills/Expertise** |
| --- | --- | --- |
| **Flight Planning & Image Acquisition** | Automated flying: Drone Deploy, Map Pilot Pro, DJI Pilot, Standardised manual flying | Piloting skills, flight mission planning, overlap calculation, accurate GSD estimation, sensors technology familiarity, handling environmental constraints |
| **Photogrammetry for Orthomosaics and 3D models creation** | Agisoft Metashape, Pix4D, Drone Deploy, Open Drone Map | Photogrammetry workflow execution, computer capacity or internet requirements, quality control of outputs, data management |
| **Image Stitching for Panorama creation** | Image Composite Editor, Adobe Photoshop, GIMP | Manual image stitching, data management |
| **GIS-Based Analysis** | QGIS, ArcGIS | GIS georeferencing, creating grids, vector creation and editing by manual annotation to count individuals, spatial data extraction, handling geospatial formats (GeoTIFF) |
| **Non-GIS Image Analysis** | ImageJ | Grid creation, manual annotation to count individuals |

# Table S3. Combinations of drone horizontal speed and camera shutter speed for different values of Ground Sampling Distance (GSD). The motion blur (in image pixels) was obtained by calculating the amount of movement the drone underwent during the exposure time (shift). A good-quality image should have a value of less than one pixel (px) of motion blur (green cells are recommended; Hann[,](https://www.ntnu.no/blogger/richard-hann/2021/10/07/preventing-motion-blur-in-drone-mapping/) 2021).

# See the link for motion blur value calculation: <https://docs.google.com/spreadsheets/d/1AmQ9guLRYLwux3mi6OCuJU4SQEiZNZhNe6maoXNK5ws/edit?gid=1692279296#gid=1692279296>

| **Drone Speed [m/s]** | **Exposure Time [s]** | **Shift (px) for GSD:0.3 cm/px** | **Shift (px) for GSD: 0.5 cm/px** | **Shift (px) for GSD: 0.7 cm/px** | **Shift (px) for GSD: 0.9 cm/px** | **Shift for GSD: 1.1 cm/px** |
| --- | --- | --- | --- | --- | --- | --- |
| 1 | 1/2000 | 0.2 | 0.1 | 0.1 | 0.1 | 0.0 |
| 1 | 1/1000 | 0.3 | 0.2 | 0.1 | 0.1 | 0.1 |
| 1 | 1/500 | 0.7 | 0.4 | 0.3 | 0.2 | 0.2 |
| 1 | 1/250 | 1.3 | 0.8 | 0.6 | 0.4 | 0.4 |
| 1 | 1/125 | 2.7 | 1.6 | 1.1 | 0.9 | 0.7 |
| 2 | 1/2000 | 0.3 | 0.2 | 0.1 | 0.1 | 0.1 |
| 2 | 1/1000 | 0.7 | 0.4 | 0.3 | 0.2 | 0.2 |
| 2 | 1/500 | 1.3 | 0.8 | 0.6 | 0.4 | 0.4 |
| 2 | 1/250 | 2.7 | 1.6 | 1.1 | 0.9 | 0.7 |
| 2 | 1/125 | 5.3 | 3.2 | 2.3 | 1.8 | 1.5 |
| 3 | 1/2000 | 0.5 | 0.3 | 0.2 | 0.2 | 0.1 |
| 3 | 1/1000 | 1.0 | 0.6 | 0.4 | 0.3 | 0.3 |
| 3 | 1/500 | 2.0 | 1.2 | 0.9 | 0.7 | 0.5 |
| 3 | 1/250 | 4.0 | 2.4 | 1.7 | 1.3 | 1.1 |
| 3 | 1/125 | 8.0 | 4.8 | 3.4 | 2.7 | 2.2 |
| 4 | 1/2000 | 0.7 | 0.4 | 0.3 | 0.2 | 0.2 |
| 4 | 1/1000 | 1.3 | 0.8 | 0.6 | 0.4 | 0.4 |
| 4 | 1/500 | 2.7 | 1.6 | 1.1 | 0.9 | 0.7 |
| 4 | 1/250 | 5.3 | 3.2 | 2.3 | 1.8 | 1.5 |
| 4 | 1/125 | 10.7 | 6.4 | 4.6 | 3.6 | 2.9 |
| 5 | 1/2000 | 0.8 | 0.5 | 0.4 | 0.3 | 0.2 |
| 5 | 1/1000 | 1.7 | 1.0 | 0.7 | 0.6 | 0.5 |
| 5 | 1/500 | 3.3 | 2.0 | 1.4 | 1.1 | 0.9 |
| 5 | 1/250 | 6.7 | 4.0 | 2.9 | 2.2 | 1.8 |
| 5 | 1/125 | 13.3 | 8.0 | 5.7 | 4.4 | 3.6 |

# Table S4. Different frontal overlap percentages based on several combinations of drone speed and height, and the image capture time interval. The overlap percentage was calculated using the short edge of the sensor, which is 3648px, and the distance in pixels that the drone moves between shots. The green cells represent overlap percentages above 75%.

# See the values calculation here: <https://docs.google.com/spreadsheets/d/1AmQ9guLRYLwux3mi6OCuJU4SQEiZNZhNe6maoXNK5ws/edit?usp=sharing>. Note that this calculation is based on the image size of the Mavic 2 Pro: 5472x3648 pixels.

| **Speed [m/s] \ Interval [s], 25m height, GSD: 0.7 cm/px** | **1 s** | **2 s** | **3 s** | **4 s** | **5 s** |
| --- | --- | --- | --- | --- | --- |
| **1 m/s** | 96.08% | 92.17% | 88.25% | 84.34% | 80.42% |
| **2 m/s** | 92.17% | 84.34% | 76.50% | 68.67% | 60.84% |
| **3 m/s** | 88.25% | 76.50% | 64.76% | 53.01% | 41.26% |
| **4 m/s** | 84.34% | 68.67% | 53.01% | 37.34% | 21.68% |
| **5 m/s** | 80.42% | 60.84% | 41.26% | 21.68% | 2.10% |
| **6 m/s** | 76.50% | 53.01% | 29.51% | 6.02% | 0.00% |
| **7 m/s** | 72.59% | 45.18% | 17.76% | 0.00% | 0.00% |
|  |  |  |  |  |  |
| **Speed [m/s] \ Interval [s], 18m height, GSD: 0.5 cm/px** | **1 s** | **2 s** | **3 s** | **4 s** | **5 s** |
| **1 m/s** | 94.52% | 89.04% | 83.55% | 78.07% | 72.59% |
| **2 m/s** | 89.04% | 78.07% | 67.11% | 56.14% | 45.18% |
| **3 m/s** | 83.55% | 67.11% | 50.66% | 34.21% | 17.76% |
| **4 m/s** | 78.07% | 56.14% | 34.21% | 12.28% | 0.00% |
| **5 m/s** | 72.59% | 45.18% | 17.76% | 0.00% | 0.00% |
| **6 m/s** | 67.11% | 34.21% | 1.32% | 0.00% | 0.00% |
| **7 m/s** | 61.62% | 23.25% | 0.00% | 0.00% | 0.00% |
|  |  |  |  |  |  |
| **Speed [m/s] \ Interval [s],**  **11m height, GSD: 0.3 cm/px** | **1 s** | **2 s** | **3 s** | **4 s** | **5 s** |
| **1 m/s** | 90.86% | 81.73% | 72.59% | 63.45% | 54.31% |
| **2 m/s** | 81.73% | 63.45% | 45.18% | 26.90% | 8.63% |
| **3 m/s** | 72.59% | 45.18% | 17.76% | 0.00% | 0.00% |
| **4 m/s** | 63.45% | 26.90% | 0.00% | 0.00% | 0.00% |
| **5 m/s** | 54.31% | 8.63% | 0.00% | 0.00% | 0.00% |
| **6 m/s** | 45.18% | 0.00% | 0.00% | 0.00% | 0.00% |
| **7 m/s** | 36.04% | 0.00% | 0.00% | 0.00% | 0.00% |

# Table S5. Different side overlap percentages based on several combinations of drone speed and height, and the image capture time interval. The overlap percentage was calculated using the long edge of the sensor, which is 5472px, and the distance in pixels that the drone moves between shots. The green cells represent overlap percentages above 75%.

# See the values calculation here: <https://docs.google.com/spreadsheets/d/1AmQ9guLRYLwux3mi6OCuJU4SQEiZNZhNe6maoXNK5ws/edit?usp=sharing>. Note that this calculation is based on the image size of the Mavic 2 Pro: 5472x3648 pixels.

| **Speed [m/s] \ Interval [s], 25m height, GSD: 0.7 cm/px** | **1 s** | **2 s** | **3 s** | **4 s** | **5 s** |
| --- | --- | --- | --- | --- | --- |
| **2 m/s** | 94.78% | 89.56% | 84.34% | 79.11% | 73.89% |
| **3 m/s** | 92.17% | 84.34% | 76.50% | 68.67% | 60.84% |
| **4 m/s** | 89.56% | 79.11% | 68.67% | 58.23% | 47.79% |
| **5 m/s** | 86.95% | 73.89% | 60.84% | 47.79% | 34.73% |
| **6 m/s** | 84.34% | 68.67% | 53.01% | 37.34% | 21.68% |
| **7 m/s** | 81.73% | 63.45% | 45.18% | 26.90% | 8.63% |
|  |  |  |  |  |  |
| **Speed [m/s] \ Interval [s], 18m height, GSD: 0.5 cm/px** | **1 s** | **2 s** | **3 s** | **4 s** | **5 s** |
| **2 m/s** | 92.69% | 85.38% | 78.07% | 70.76% | 63.45% |
| **3 m/s** | 89.04% | 78.07% | 67.11% | 56.14% | 45.18% |
| **4 m/s** | 85.38% | 70.76% | 56.14% | 41.52% | 26.90% |
| **5 m/s** | 81.73% | 63.45% | 45.18% | 26.90% | 8.63% |
| **6 m/s** | 78.07% | 56.14% | 34.21% | 12.28% | 0.00% |
| **7 m/s** | 74.42% | 48.83% | 23.25% | 0.00% | 0.00% |
|  |  |  |  |  |  |
| **Speed [m/s] \ Interval [s], 11m height, GSD 0.3 cm/px** | **1 s** | **2 s** | **3 s** | **4 s** | **5 s** |
| **2 m/s** | 87.82% | 75.63% | 63.45% | 51.27% | 39.08% |
| **3 m/s** | 81.73% | 63.45% | 45.18% | 26.90% | 8.63% |
| **4 m/s** | 75.63% | 51.27% | 26.90% | 2.53% | 0.00% |
| **5 m/s** | 69.54% | 39.08% | 8.63% | 0.00% | 0.00% |
| **6 m/s** | 63.45% | 26.90% | 0.00% | 0.00% | 0.00% |
| **7 m/s** | 57.36% | 14.72% | 0.00% | 0.00% | 0.00% |

# Table S6. Comparison of counts from four experts to assess their similarity in a dataset of 465 aerial images. The column "Iguana presence" represents the consensus response among the experts, with three out of four agreeing that the image does or does not contain iguana(s). We calculated the coefficient of variation (CV) among counts for every image to compare variability across datasets: CV = (Standard deviation / Mean) × 100.

| **Image name** | **Exp1** | **Exp2** | **Exp 3** | **Exp 4** | **Iguana presence** | **CV (%)** |
| --- | --- | --- | --- | --- | --- | --- |
| Fer_FPE03-04-05_18122021_93_23.jpg | 2 | 1 | 5 | 1 | Yes | 84.13 |
| Fer_FCD01-02-03_20122021_6_43.jpg | 1 | 0 | 1 | 2 | Yes | 81.65 |
| Fer_FCD01-02-03_20122021_47_26.jpg | 2 | 1 | 1 | 0 | Yes | 81.65 |
| Fer_FEA02-03_18122021_63_33.jpg | 2 | 1 | 0 | 1 | Yes | 81.65 |
| Isa_ISTI01-02-03_27012023_45_33.jpg | 2 | 1 | 0 | 1 | Yes | 81.65 |
| Isa_ISVB03_77012023_26_23.jpg | 2 | 1 | 0 | 2 | Yes | 76.59 |
| Isa_ISTI01-02-03_27012023_19_24.jpg | 1 | 1 | 1 | 3 | Yes | 66.67 |
| Fer_FCD01-02-03_20122021_80_3.jpg | 1 | 1 | 1 | 0 | Yes | 66.67 |
| Fer_FEA01_18122021_12_15.jpg | 1 | 1 | 1 | 0 | Yes | 66.67 |
| Fer_FPM01-02_20012023_69_17.jpg | 1 | 1 | 1 | 0 | Yes | 66.67 |
| Fer_FCD01-02-03_20122021_16_21.jpg | 1 | 1 | 0 | 1 | Yes | 66.67 |
| Fer_FEA02-03_18122021_44_22.jpg | 1 | 1 | 0 | 1 | Yes | 66.67 |
| Isa_ISTI01-02-03_27012023_3_44.jpg | 1 | 1 | 0 | 1 | Yes | 66.67 |
| Fer_FCD01-02-03_20122021_13_8.jpg | 1 | 0 | 1 | 1 | Yes | 66.67 |
| Fer_FCD01-02-03_20122021_56_13.jpg | 1 | 0 | 1 | 1 | Yes | 66.67 |
| Isa_ISVB01_27012023_49_39.jpg | 2 | 0 | 2 | 2 | Yes | 66.67 |
| Fer_FEA02-03_18122021_24_16.jpg | 1 | 1 | 2 | 1 | Yes | 40.00 |
| Fer_FCD01-02-03_20122021_52_24.jpg | 1 | 1 | 1 | 2 | Yes | 40.00 |
| Isa_ISVB01_27012023_50_39.jpg | 1 | 1 | 1 | 2 | Yes | 40.00 |
| Fer_FCD01-02-03_20122021_51_26.jpg | 2 | 1 | 1 | 1 | Yes | 40.00 |
| Isa_ISCWN02_18012023_80_35.jpg | 2 | 1 | 1 | 1 | Yes | 40.00 |
| Isa_ISTI01-02-03_27012023_13_50.jpg | 3 | 3 | 3 | 1 | Yes | 40.00 |
| Fer_FPE01-06_18122021_35_24.jpg | 2 | 2 | 1 | 1 | Yes | 38.49 |
| Fer_FPM01-02_20012023_50_18.jpg | 2 | 2 | 1 | 1 | Yes | 38.49 |
| Fer_FPE01-06_18122021_38_28.jpg | 6 | 4 | 4 | 3 | Yes | 29.61 |
| Fer_FPM01-02_20012023_27_109.jpg | 3 | 3 | 2 | 2 | Yes | 23.09 |
| Fer_FCD01-02-03_20122021_34_35.jpg | 2 | 2 | 3 | 2 | Yes | 22.22 |
| Fer_FPM01-02_20012023_18_115.jpg | 2 | 2 | 2 | 3 | Yes | 22.22 |
| Fer_FCD01-02-03_20122021_12_16.jpg | 3 | 2 | 2 | 2 | Yes | 22.22 |
| Isa_ISVB02_27012023_61.jpg | 3 | 2 | 2 | 2 | Yes | 22.22 |
| Fer_FPM01-02_20012023_50_97.jpg | 4 | 4 | 5 | 3 | Yes | 20.41 |
| Fer_FPM01-02_20012023_56_54.jpg | 3 | 3 | 3 | 2 | Yes | 18.18 |
| Isa_ISVB03_77012023_25_22.jpg | 3 | 3 | 3 | 2 | Yes | 18.18 |
| Isa_ISVB03_77012023_28_18.jpg | 3 | 3 | 4 | 4 | Yes | 16.50 |
| Isa_ISVB01_27012023_73_40.jpg | 9 | 10 | 11 | 8 | Yes | 13.59 |
| Isa_ISTI01-02-03_27012023_2_45.jpg | 4 | 4 | 4 | 3 | Yes | 13.33 |
| Isa_ISVB01_27012023_74_43.jpg | 4 | 3 | 4 | 4 | Yes | 13.33 |
| Fer_FPM01-02_20012023_32_10.jpg | 5 | 4 | 5 | 4 | Yes | 12.83 |
| Fer_FPE01-06_18122021_31_27.jpg | 9 | 7 | 8 | 7 | Yes | 12.35 |
| Fer_FPM01-02_20012023_53_104.jpg | 4 | 4 | 5 | 4 | Yes | 11.76 |
| Isa_ISTI01-02-03_27012023_6_40.jpg | 9 | 7 | 8 | 8 | Yes | 10.21 |
| Fer_FCD01-02-03_20122021_1_43.jpg | 41 | 37 | 34 | 33 | Yes | 9.91 |
| Fer_FEA02-03_18122021_20_16 | 23 | 24 | 28 | 23 | Yes | 9.72 |
| Fer_FCD01-02-03_20122021_28_27.jpg | 10 | 10 | 10 | 12 | Yes | 9.52 |
| Fer_FCD01-02-03_20122021_31_22.jpg | 11 | 10 | 10 | 9 | Yes | 8.16 |
| Fer_FPM01-02_20012023_43_79.jpg | 73 | 74 | 86 | 75 | Yes | 7.86 |
| Fer_FCD01-02-03_20122021_10_23.jpg | 100 | 104 | 91 | 90 | Yes | 7.12 |
| Fer_FCD01-02-03_20122021_47_32.jpg | 9 | 9 | 8 | 8 | Yes | 6.79 |
| Fer_FPM01-02_20012023_11_115.jpg | 25 | 25 | 24 | 22 | Yes | 5.89 |
| Fer_FCD01-02-03_20122021_16_15.jpg | 62 | 62 | 66 | 58 | Yes | 5.27 |
| Fer_FEA01_18122021_3_16 | 43 | 44 | 43 | 39 | Yes | 5.25 |
| Isa_ISCWN02_18012023_108_19.jpg | 21 | 21 | 21 | 19 | Yes | 4.88 |
| Fer_FCD01-02-03_20122021_27_28.jpg | 29 | 30 | 29 | 32 | Yes | 4.71 |
| Fer_FCD01-02-03_20122021_28_19.jpg | 11 | 11 | 10 | 11 | Yes | 4.65 |
| Fer_FCD01-02-03_20122021_80_4.jpg | 43 | 41 | 39 | 43 | Yes | 4.61 |
| Fer_FEA01_18122021_5_6 | 45 | 43 | 41 | 42 | Yes | 3.99 |
| Fer_FPM01-02_20012023_41_78.jpg | 15 | 14 | 14 | 14 | Yes | 3.51 |
| Fer_FPE03-04-05_18122021_99_18.jpg | 18 | 17 | 18 | 17 | Yes | 3.30 |
| Fer_FCD01-02-03_20122021_14_14.jpg | 16 | 17 | 16 | 16 | Yes | 3.08 |
| Fer_FPM01-02_20012023_56_79.jpg | 17 | 17 | 17 | 16 | Yes | 2.99 |
| Fer_FPE03-04-05_18122021_98_19.jpg | 50 | 52 | 51 | 49 | Yes | 2.56 |
| Fer_FEA01_18122021_7_13 | 59 | 58 | 61 | 58 | Yes | 2.40 |
| Fer_FEA02-03_18122021_20_15.jpg | 45 | 45 | 47 | 45 | Yes | 2.20 |
| Fer_FCD01-02-03_20122021_15_14.jpg | 99 | 95 | 98 | 95 | Yes | 2.13 |
| Fer_FCD01-02-03_20122021_18_21.jpg | 0 | 0 | 0 | 0 | No | 0.00 |
| Fer_FCD01-02-03_20122021_20_14.jpg | 0 | 0 | 0 | 0 | No | 0.00 |
| Fer_FCD01-02-03_20122021_20_16.jpg | 0 | 0 | 0 | 0 | No | 0.00 |
| Fer_FCD01-02-03_20122021_22_15.jpg | 0 | 0 | 0 | 0 | No | 0.00 |
| Fer_FCD01-02-03_20122021_27_25.jpg | 0 | 0 | 0 | 0 | No | 0.00 |
| Fer_FCD01-02-03_20122021_3_21.jpg | 0 | 0 | 0 | 0 | No | 0.00 |
| Fer_FCD01-02-03_20122021_30_36.jpg | 0 | 0 | 0 | 0 | No | 0.00 |
| Fer_FCD01-02-03_20122021_30_37.jpg | 0 | 0 | 0 | 0 | No | 0.00 |
| Fer_FCD01-02-03_20122021_31_41.jpg | 0 | 0 | 0 | 0 | No | 0.00 |
| Fer_FCD01-02-03_20122021_32_37.jpg | 0 | 0 | 0 | 0 | No | 0.00 |
| Fer_FCD01-02-03_20122021_32_42.jpg | 0 | 0 | 0 | 0 | No | 0.00 |
| Fer_FCD01-02-03_20122021_4_47.jpg | 0 | 0 | 0 | 0 | No | 0.00 |
| Fer_FCD01-02-03_20122021_42_37.jpg | 0 | 0 | 0 | 0 | No | 0.00 |
| Fer_FCD01-02-03_20122021_51_35.jpg | 0 | 0 | 0 | 0 | No | 0.00 |
| Fer_FCD01-02-03_20122021_53_17.jpg | 0 | 0 | 0 | 0 | No | 0.00 |
| Fer_FCD01-02-03_20122021_53_29.jpg | 0 | 0 | 0 | 0 | No | 0.00 |
| Fer_FCD01-02-03_20122021_61_15.jpg | 0 | 0 | 0 | 0 | No | 0.00 |
| Fer_FCD01-02-03_20122021_66_13.jpg | 0 | 0 | 0 | 0 | No | 0.00 |
| Fer_FCD01-02-03_20122021_68_13.jpg | 0 | 0 | 0 | 0 | No | 0.00 |
| Fer_FCD01-02-03_20122021_77_10.jpg | 0 | 0 | 0 | 0 | No | 0.00 |
| Fer_FCD01-02-03_20122021_78_8.jpg | 0 | 0 | 0 | 0 | No | 0.00 |
| Fer_FCD01-02-03_20122021_8_20.jpg | 0 | 0 | 0 | 0 | No | 0.00 |
| Fer_FCD01-02-03_20122021_83_7.jpg | 0 | 0 | 0 | 0 | No | 0.00 |
| Fer_FCD01-02-03_20122021_85_4.jpg | 0 | 0 | 0 | 0 | No | 0.00 |
| Fer_FEA01_18122021_12_5.jpg | 0 | 0 | 0 | 0 | No | 0.00 |
| Fer_FEA01_18122021_14_22.jpg | 0 | 0 | 0 | 0 | No | 0.00 |
| Fer_FEA01_18122021_25_25.jpg | 0 | 0 | 0 | 0 | No | 0.00 |
| Fer_FEA01_18122021_26_37.jpg | 0 | 0 | 0 | 0 | No | 0.00 |
| Fer_FEA01_18122021_27_34.jpg | 0 | 0 | 0 | 0 | No | 0.00 |
| Fer_FEA01_18122021_27_36.jpg | 0 | 0 | 0 | 0 | No | 0.00 |
| Fer_FEA01_18122021_38_42.jpg | 0 | 0 | 0 | 0 | No | 0.00 |
| Fer_FEA01_18122021_45_43.jpg | 0 | 0 | 0 | 0 | No | 0.00 |
| Fer_FEA01_18122021_46_40.jpg | 0 | 0 | 0 | 0 | No | 0.00 |
| Fer_FEA01_18122021_46_42.jpg | 0 | 0 | 0 | 0 | No | 0.00 |
| Fer_FEA01_18122021_5_17.jpg | 0 | 0 | 0 | 0 | No | 0.00 |
| Fer_FEA01_18122021_5_20.jpg | 0 | 0 | 0 | 0 | No | 0.00 |
| Fer_FEA01_18122021_6_15.jpg | 0 | 0 | 0 | 0 | No | 0.00 |
| Fer_FEA01_18122021_6_22.jpg | 0 | 0 | 0 | 0 | No | 0.00 |
| Fer_FEA01_18122021_8_24.jpg | 0 | 0 | 0 | 0 | No | 0.00 |
| Fer_FEA01_18122021_9_2.jpg | 0 | 0 | 0 | 0 | No | 0.00 |
| Fer_FEA02-03_18122021_11_21.jpg | 0 | 0 | 0 | 0 | No | 0.00 |
| Fer_FEA02-03_18122021_2_12.jpg | 0 | 0 | 0 | 0 | No | 0.00 |
| Fer_FEA02-03_18122021_21_23 | 0 | 0 | 0 | 0 | No | 0.00 |
| Fer_FEA02-03_18122021_25_21.jpg | 0 | 0 | 0 | 0 | No | 0.00 |
| Fer_FEA02-03_18122021_31_18.jpg | 0 | 0 | 0 | 0 | No | 0.00 |
| Fer_FEA02-03_18122021_31_20.jpg | 0 | 0 | 0 | 0 | No | 0.00 |
| Fer_FEA02-03_18122021_36_13.jpg | 0 | 0 | 0 | 0 | No | 0.00 |
| Fer_FEA02-03_18122021_38_18.jpg | 0 | 0 | 0 | 0 | No | 0.00 |
| Fer_FEA02-03_18122021_38_20.jpg | 0 | 0 | 0 | 0 | No | 0.00 |
| Fer_FEA02-03_18122021_39_11.jpg | 0 | 0 | 0 | 0 | No | 0.00 |
| Fer_FEA02-03_18122021_39_21.jpg | 0 | 0 | 0 | 0 | No | 0.00 |
| Fer_FEA02-03_18122021_39_24.jpg | 0 | 0 | 0 | 0 | No | 0.00 |
| Fer_FEA02-03_18122021_40_23.jpg | 0 | 0 | 0 | 0 | No | 0.00 |
| Fer_FEA02-03_18122021_42_7.jpg | 0 | 0 | 0 | 0 | No | 0.00 |
| Fer_FEA02-03_18122021_43_21.jpg | 0 | 0 | 0 | 0 | No | 0.00 |
| Fer_FEA02-03_18122021_44_24.jpg | 0 | 0 | 0 | 0 | No | 0.00 |
| Fer_FEA02-03_18122021_45_24.jpg | 0 | 0 | 0 | 0 | No | 0.00 |
| Fer_FEA02-03_18122021_46_14.jpg | 0 | 0 | 0 | 0 | No | 0.00 |
| Fer_FEA02-03_18122021_47_10.jpg | 0 | 0 | 0 | 0 | No | 0.00 |
| Fer_FEA02-03_18122021_47_20.jpg | 0 | 0 | 0 | 0 | No | 0.00 |
| Fer_FEA02-03_18122021_48_30.jpg | 0 | 0 | 0 | 0 | No | 0.00 |
| Fer_FEA02-03_18122021_49_28.jpg | 0 | 0 | 0 | 0 | No | 0.00 |
| Fer_FEA02-03_18122021_50_25.jpg | 0 | 0 | 0 | 0 | No | 0.00 |
| Fer_FEA02-03_18122021_50_29.jpg | 0 | 0 | 0 | 0 | No | 0.00 |
| Fer_FEA02-03_18122021_51_15.jpg | 0 | 0 | 0 | 0 | No | 0.00 |
| Fer_FEA02-03_18122021_53_31.jpg | 0 | 0 | 0 | 0 | No | 0.00 |
| Fer_FEA02-03_18122021_55_28.jpg | 0 | 0 | 0 | 0 | No | 0.00 |
| Fer_FEA02-03_18122021_6_15.jpg | 0 | 0 | 0 | 0 | No | 0.00 |
| Fer_FEA02-03_18122021_61_34.jpg | 0 | 0 | 0 | 0 | No | 0.00 |
| Fer_FEA02-03_18122021_7_19.jpg | 0 | 0 | 0 | 0 | No | 0.00 |
| Fer_FEA02-03_18122021_72_39.jpg | 0 | 0 | 0 | 0 | No | 0.00 |
| Fer_FEA02-03_18122021_72_42.jpg | 0 | 0 | 0 | 0 | No | 0.00 |
| Fer_FEA02-03_18122021_8_24.jpg | 0 | 0 | 0 | 0 | No | 0.00 |
| Fer_FPE01-06_18122021_11_49.jpg | 0 | 0 | 0 | 0 | No | 0.00 |
| Fer_FPE01-06_18122021_24_50.jpg | 0 | 0 | 0 | 0 | No | 0.00 |
| Fer_FPE01-06_18122021_25_33.jpg | 0 | 0 | 0 | 0 | No | 0.00 |
| Fer_FPE01-06_18122021_26_50.jpg | 0 | 0 | 0 | 0 | No | 0.00 |
| Fer_FPE01-06_18122021_31_16.jpg | 0 | 0 | 0 | 0 | No | 0.00 |
| Fer_FPE01-06_18122021_31_31.jpg | 0 | 0 | 0 | 0 | No | 0.00 |
| Fer_FPE01-06_18122021_31_32.jpg | 0 | 0 | 0 | 0 | No | 0.00 |
| Fer_FPE01-06_18122021_44_9.jpg | 0 | 0 | 0 | 0 | No | 0.00 |
| Fer_FPE01-06_18122021_46_0.jpg | 0 | 0 | 0 | 0 | No | 0.00 |
| Fer_FPE02_18122021_10_12.jpg | 0 | 0 | 0 | 0 | No | 0.00 |
| Fer_FPE02_18122021_13_5.jpg | 0 | 0 | 0 | 0 | No | 0.00 |
| Fer_FPE02_18122021_14_5.jpg | 0 | 0 | 0 | 0 | No | 0.00 |
| Fer_FPE02_18122021_17_8.jpg | 0 | 0 | 0 | 0 | No | 0.00 |
| Fer_FPE02_18122021_18_13.jpg | 0 | 0 | 0 | 0 | No | 0.00 |
| Fer_FPE02_18122021_21_3.jpg | 0 | 0 | 0 | 0 | No | 0.00 |
| Fer_FPE02_18122021_22_6.jpg | 0 | 0 | 0 | 0 | No | 0.00 |
| Fer_FPE02_18122021_26_6.jpg | 0 | 0 | 0 | 0 | No | 0.00 |
| Fer_FPE02_18122021_3_10.jpg | 0 | 0 | 0 | 0 | No | 0.00 |
| Fer_FPE02_18122021_34_12.jpg | 0 | 0 | 0 | 0 | No | 0.00 |
| Fer_FPE02_18122021_36_20.jpg | 0 | 0 | 0 | 0 | No | 0.00 |
| Fer_FPE02_18122021_5_14.jpg | 0 | 0 | 0 | 0 | No | 0.00 |
| Fer_FPE03-04-05_18122021_101_22.jpg | 0 | 0 | 0 | 0 | No | 0.00 |
| Fer_FPE03-04-05_18122021_101_24.jpg | 0 | 0 | 0 | 0 | No | 0.00 |
| Fer_FPE03-04-05_18122021_103_23.jpg | 0 | 0 | 0 | 0 | No | 0.00 |
| Fer_FPE03-04-05_18122021_109_21.jpg | 0 | 0 | 0 | 0 | No | 0.00 |
| Fer_FPE03-04-05_18122021_113_20.jpg | 0 | 0 | 0 | 0 | No | 0.00 |
| Fer_FPE03-04-05_18122021_116_9.jpg | 0 | 0 | 0 | 0 | No | 0.00 |
| Fer_FPE03-04-05_18122021_119_6.jpg | 0 | 0 | 0 | 0 | No | 0.00 |
| Fer_FPE03-04-05_18122021_13_14.jpg | 0 | 0 | 0 | 0 | No | 0.00 |
| Fer_FPE03-04-05_18122021_33_8.jpg | 0 | 0 | 0 | 0 | No | 0.00 |
| Fer_FPE03-04-05_18122021_37_4.jpg | 0 | 0 | 0 | 0 | No | 0.00 |
| Fer_FPE03-04-05_18122021_41_10.jpg | 0 | 0 | 0 | 0 | No | 0.00 |
| Fer_FPE03-04-05_18122021_44_8.jpg | 0 | 0 | 0 | 0 | No | 0.00 |
| Fer_FPE03-04-05_18122021_51_16.jpg | 0 | 0 | 0 | 0 | No | 0.00 |
| Fer_FPE03-04-05_18122021_52_16.jpg | 0 | 0 | 0 | 0 | No | 0.00 |
| Fer_FPE03-04-05_18122021_55_26.jpg | 0 | 0 | 0 | 0 | No | 0.00 |
| Fer_FPE03-04-05_18122021_56_27.jpg | 0 | 0 | 0 | 0 | No | 0.00 |
| Fer_FPE03-04-05_18122021_58_33.jpg | 0 | 0 | 0 | 0 | No | 0.00 |
| Fer_FPE03-04-05_18122021_65_16.jpg | 0 | 0 | 0 | 0 | No | 0.00 |
| Fer_FPE03-04-05_18122021_67_23.jpg | 0 | 0 | 0 | 0 | No | 0.00 |
| Fer_FPE03-04-05_18122021_69_18.jpg | 0 | 0 | 0 | 0 | No | 0.00 |
| Fer_FPE03-04-05_18122021_73_26.jpg | 0 | 0 | 0 | 0 | No | 0.00 |
| Fer_FPE03-04-05_18122021_75_17.jpg | 0 | 0 | 0 | 0 | No | 0.00 |
| Fer_FPE03-04-05_18122021_98_24.jpg | 0 | 0 | 0 | 0 | No | 0.00 |
| Fer_FPM01-02_20012023_14_116.jpg | 0 | 0 | 0 | 0 | No | 0.00 |
| Fer_FPM01-02_20012023_15_118.jpg | 0 | 0 | 0 | 0 | No | 0.00 |
| Fer_FPM01-02_20012023_17_9.jpg | 0 | 0 | 0 | 0 | No | 0.00 |
| Fer_FPM01-02_20012023_19_14.jpg | 0 | 0 | 0 | 0 | No | 0.00 |
| Fer_FPM01-02_20012023_21_106.jpg | 0 | 0 | 0 | 0 | No | 0.00 |
| Fer_FPM01-02_20012023_24_116.jpg | 0 | 0 | 0 | 0 | No | 0.00 |
| Fer_FPM01-02_20012023_27_117.jpg | 0 | 0 | 0 | 0 | No | 0.00 |
| Fer_FPM01-02_20012023_29_8.jpg | 0 | 0 | 0 | 0 | No | 0.00 |
| Fer_FPM01-02_20012023_31_98.jpg | 0 | 0 | 0 | 0 | No | 0.00 |
| Fer_FPM01-02_20012023_32_126.jpg | 0 | 0 | 0 | 0 | No | 0.00 |
| Fer_FPM01-02_20012023_34_105.jpg | 0 | 0 | 0 | 0 | No | 0.00 |
| Fer_FPM01-02_20012023_34_95.jpg | 0 | 0 | 0 | 0 | No | 0.00 |
| Fer_FPM01-02_20012023_39_68.jpg | 0 | 0 | 0 | 0 | No | 0.00 |
| Fer_FPM01-02_20012023_39_70.jpg | 0 | 0 | 0 | 0 | No | 0.00 |
| Fer_FPM01-02_20012023_4_125.jpg | 0 | 0 | 0 | 0 | No | 0.00 |
| Fer_FPM01-02_20012023_40_87.jpg | 0 | 0 | 0 | 0 | No | 0.00 |
| Fer_FPM01-02_20012023_41_10.jpg | 0 | 0 | 0 | 0 | No | 0.00 |
| Fer_FPM01-02_20012023_41_85.jpg | 0 | 0 | 0 | 0 | No | 0.00 |
| Fer_FPM01-02_20012023_41_91.jpg | 0 | 0 | 0 | 0 | No | 0.00 |
| Fer_FPM01-02_20012023_43_7.jpg | 0 | 0 | 0 | 0 | No | 0.00 |
| Fer_FPM01-02_20012023_44_89.jpg | 0 | 0 | 0 | 0 | No | 0.00 |
| Fer_FPM01-02_20012023_45_17.jpg | 0 | 0 | 0 | 0 | No | 0.00 |
| Fer_FPM01-02_20012023_48_50.jpg | 0 | 0 | 0 | 0 | No | 0.00 |
| Fer_FPM01-02_20012023_48_61.jpg | 0 | 0 | 0 | 0 | No | 0.00 |
| Fer_FPM01-02_20012023_49_56.jpg | 0 | 0 | 0 | 0 | No | 0.00 |
| Fer_FPM01-02_20012023_5_120.jpg | 0 | 0 | 0 | 0 | No | 0.00 |
| Fer_FPM01-02_20012023_50_68.jpg | 0 | 0 | 0 | 0 | No | 0.00 |
| Fer_FPM01-02_20012023_51_71.jpg | 0 | 0 | 0 | 0 | No | 0.00 |
| Fer_FPM01-02_20012023_52_3.jpg | 0 | 0 | 0 | 0 | No | 0.00 |
| Fer_FPM01-02_20012023_53_101.jpg | 0 | 0 | 0 | 0 | No | 0.00 |
| Fer_FPM01-02_20012023_53_70.jpg | 0 | 0 | 0 | 0 | No | 0.00 |
| Fer_FPM01-02_20012023_57_64.jpg | 0 | 0 | 0 | 0 | No | 0.00 |
| Fer_FPM01-02_20012023_59_65.jpg | 0 | 0 | 0 | 0 | No | 0.00 |
| Fer_FPM01-02_20012023_66_19.jpg | 0 | 0 | 0 | 0 | No | 0.00 |
| Isa_ISCAS01_08012023_10_28.jpg | 0 | 0 | 0 | 0 | No | 0.00 |
| Isa_ISCAS01_08012023_41_28.jpg | 0 | 0 | 0 | 0 | No | 0.00 |
| Isa_ISCAS01_08012023_42_31.jpg | 0 | 0 | 0 | 0 | No | 0.00 |
| Isa_ISCAS01_08012023_46_28.jpg | 0 | 0 | 0 | 0 | No | 0.00 |
| Isa_ISCAS01_08012023_47_27.jpg | 0 | 0 | 0 | 0 | No | 0.00 |
| Isa_ISCAS01_08012023_49_26.jpg | 0 | 0 | 0 | 0 | No | 0.00 |
| Isa_ISCAS01_08012023_55_29.jpg | 0 | 0 | 0 | 0 | No | 0.00 |
| Isa_ISCAS01_08012023_58_18.jpg | 0 | 0 | 0 | 0 | No | 0.00 |
| Isa_ISCAS01_08012023_58_19.jpg | 0 | 0 | 0 | 0 | No | 0.00 |
| Isa_ISCAS01_08012023_60_19.jpg | 0 | 0 | 0 | 0 | No | 0.00 |
| Isa_ISCAS01_08012023_64_16.jpg | 0 | 0 | 0 | 0 | No | 0.00 |
| Isa_ISCAS01_08012023_7_29.jpg | 0 | 0 | 0 | 0 | No | 0.00 |
| Isa_ISCAS01_08012023_82_1.jpg | 0 | 0 | 0 | 0 | No | 0.00 |
| Isa_ISCAS02_08012023_10_11.jpg | 0 | 0 | 0 | 0 | No | 0.00 |
| Isa_ISCAS02_08012023_10_13.jpg | 0 | 0 | 0 | 0 | No | 0.00 |
| Isa_ISCAS02_08012023_12_14.jpg | 0 | 0 | 0 | 0 | No | 0.00 |
| Isa_ISCAS02_08012023_16_11.jpg | 0 | 0 | 0 | 0 | No | 0.00 |
| Isa_ISCAS02_08012023_16_16.jpg | 0 | 0 | 0 | 0 | No | 0.00 |
| Isa_ISCAS02_08012023_16_17.jpg | 0 | 0 | 0 | 0 | No | 0.00 |
| Isa_ISCAS02_08012023_22_9.jpg | 0 | 0 | 0 | 0 | No | 0.00 |
| Isa_ISCAS02_08012023_3_31.jpg | 0 | 0 | 0 | 0 | No | 0.00 |
| Isa_ISCAS02_08012023_39_9.jpg | 0 | 0 | 0 | 0 | No | 0.00 |
| Isa_ISCAS02_08012023_50_9.jpg | 0 | 0 | 0 | 0 | No | 0.00 |
| Isa_ISCAS02_08012023_56_45.jpg | 0 | 0 | 0 | 0 | No | 0.00 |
| Isa_ISCAS02_08012023_57_13.jpg | 0 | 0 | 0 | 0 | No | 0.00 |
| Isa_ISCAS02_08012023_60_14.jpg | 0 | 0 | 0 | 0 | No | 0.00 |
| Isa_ISCAS02_08012023_79_74.jpg | 0 | 0 | 0 | 0 | No | 0.00 |
| Isa_ISCAS03_08012023_10_11.jpg | 0 | 0 | 0 | 0 | No | 0.00 |
| Isa_ISCAS03_08012023_11_15.jpg | 0 | 0 | 0 | 0 | No | 0.00 |
| Isa_ISCAS03_08012023_11_7.jpg | 0 | 0 | 0 | 0 | No | 0.00 |
| Isa_ISCAS03_08012023_12_10.jpg | 0 | 0 | 0 | 0 | No | 0.00 |
| Isa_ISCAS03_08012023_12_13.jpg | 0 | 0 | 0 | 0 | No | 0.00 |
| Isa_ISCAS03_08012023_12_8.jpg | 0 | 0 | 0 | 0 | No | 0.00 |
| Isa_ISCAS03_08012023_14_11.jpg | 0 | 0 | 0 | 0 | No | 0.00 |
| Isa_ISCAS03_08012023_14_13.jpg | 0 | 0 | 0 | 0 | No | 0.00 |
| Isa_ISCAS03_08012023_15_13.jpg | 0 | 0 | 0 | 0 | No | 0.00 |
| Isa_ISCAS03_08012023_15_15.jpg | 0 | 0 | 0 | 0 | No | 0.00 |
| Isa_ISCAS03_08012023_16_9.jpg | 0 | 0 | 0 | 0 | No | 0.00 |
| Isa_ISCAS03_08012023_17_12.jpg | 0 | 0 | 0 | 0 | No | 0.00 |
| Isa_ISCAS03_08012023_18_9.jpg | 0 | 0 | 0 | 0 | No | 0.00 |
| Isa_ISCAS03_08012023_19_13.jpg | 0 | 0 | 0 | 0 | No | 0.00 |
| Isa_ISCAS03_08012023_21_12.jpg | 0 | 0 | 0 | 0 | No | 0.00 |
| Isa_ISCAS03_08012023_31_13.jpg | 0 | 0 | 0 | 0 | No | 0.00 |
| Isa_ISCAS03_08012023_31_26.jpg | 0 | 0 | 0 | 0 | No | 0.00 |
| Isa_ISCAS03_08012023_32_20.jpg | 0 | 0 | 0 | 0 | No | 0.00 |
| Isa_ISCAS03_08012023_33_18.jpg | 0 | 0 | 0 | 0 | No | 0.00 |
| Isa_ISCAS03_08012023_33_21.jpg | 0 | 0 | 0 | 0 | No | 0.00 |
| Isa_ISCAS03_08012023_33_22.jpg | 0 | 0 | 0 | 0 | No | 0.00 |
| Isa_ISCAS03_08012023_35_32.jpg | 0 | 0 | 0 | 0 | No | 0.00 |
| Isa_ISCAS03_08012023_36_29.jpg | 0 | 0 | 0 | 0 | No | 0.00 |
| Isa_ISCAS03_08012023_38_24.jpg | 0 | 0 | 0 | 0 | No | 0.00 |
| Isa_ISCAS03_08012023_39_29.jpg | 0 | 0 | 0 | 0 | No | 0.00 |
| Isa_ISCAS03_08012023_42_35.jpg | 0 | 0 | 0 | 0 | No | 0.00 |
| Isa_ISCAS03_08012023_49_49.jpg | 0 | 0 | 0 | 0 | No | 0.00 |
| Isa_ISCAS03_08012023_50_39.jpg | 0 | 0 | 0 | 0 | No | 0.00 |
| Isa_ISCAS03_08012023_50_43.jpg | 0 | 0 | 0 | 0 | No | 0.00 |
| Isa_ISCAS03_08012023_50_49.jpg | 0 | 0 | 0 | 0 | No | 0.00 |
| Isa_ISCAS03_08012023_51_38.jpg | 0 | 0 | 0 | 0 | No | 0.00 |
| Isa_ISCAS03_08012023_69_41.jpg | 0 | 0 | 0 | 0 | No | 0.00 |
| Isa_ISCWN02_18012023_100_37.jpg | 0 | 0 | 0 | 0 | No | 0.00 |
| Isa_ISCWN02_18012023_100_41.jpg | 0 | 0 | 0 | 0 | No | 0.00 |
| Isa_ISCWN02_18012023_100_46.jpg | 0 | 0 | 0 | 0 | No | 0.00 |
| Isa_ISCWN02_18012023_101_35.jpg | 0 | 0 | 0 | 0 | No | 0.00 |
| Isa_ISCWN02_18012023_102_27.jpg | 0 | 0 | 0 | 0 | No | 0.00 |
| Isa_ISCWN02_18012023_102_34.jpg | 0 | 0 | 0 | 0 | No | 0.00 |
| Isa_ISCWN02_18012023_103_43.jpg | 0 | 0 | 0 | 0 | No | 0.00 |
| Isa_ISCWN02_18012023_105_26.jpg | 0 | 0 | 0 | 0 | No | 0.00 |
| Isa_ISCWN02_18012023_105_28.jpg | 0 | 0 | 0 | 0 | No | 0.00 |
| Isa_ISCWN02_18012023_106_23.jpg | 0 | 0 | 0 | 0 | No | 0.00 |
| Isa_ISCWN02_18012023_107_25.jpg | 0 | 0 | 0 | 0 | No | 0.00 |
| Isa_ISCWN02_18012023_108_26.jpg | 0 | 0 | 0 | 0 | No | 0.00 |
| Isa_ISCWN02_18012023_108_28.jpg | 0 | 0 | 0 | 0 | No | 0.00 |
| Isa_ISCWN02_18012023_108_29.jpg | 0 | 0 | 0 | 0 | No | 0.00 |
| Isa_ISCWN02_18012023_11_47.jpg | 0 | 0 | 0 | 0 | No | 0.00 |
| Isa_ISCWN02_18012023_11_52.jpg | 0 | 0 | 0 | 0 | No | 0.00 |
| Isa_ISCWN02_18012023_112_22.jpg | 0 | 0 | 0 | 0 | No | 0.00 |
| Isa_ISCWN02_18012023_113_18.jpg | 0 | 0 | 0 | 0 | No | 0.00 |
| Isa_ISCWN02_18012023_12_47.jpg | 0 | 0 | 0 | 0 | No | 0.00 |
| Isa_ISCWN02_18012023_122_23.jpg | 0 | 0 | 0 | 0 | No | 0.00 |
| Isa_ISCWN02_18012023_127_17.jpg | 0 | 0 | 0 | 0 | No | 0.00 |
| Isa_ISCWN02_18012023_128_15.jpg | 0 | 0 | 0 | 0 | No | 0.00 |
| Isa_ISCWN02_18012023_128_18.jpg | 0 | 0 | 0 | 0 | No | 0.00 |
| Isa_ISCWN02_18012023_129_15.jpg | 0 | 0 | 0 | 0 | No | 0.00 |
| Isa_ISCWN02_18012023_129_8.jpg | 0 | 0 | 0 | 0 | No | 0.00 |
| Isa_ISCWN02_18012023_18_44.jpg | 0 | 0 | 0 | 0 | No | 0.00 |
| Isa_ISCWN02_18012023_19_43.jpg | 0 | 0 | 0 | 0 | No | 0.00 |
| Isa_ISCWN02_18012023_20_42.jpg | 0 | 0 | 0 | 0 | No | 0.00 |
| Isa_ISCWN02_18012023_20_43.jpg | 0 | 0 | 0 | 0 | No | 0.00 |
| Isa_ISCWN02_18012023_23_34.jpg | 0 | 0 | 0 | 0 | No | 0.00 |
| Isa_ISCWN02_18012023_23_38.jpg | 0 | 0 | 0 | 0 | No | 0.00 |
| Isa_ISCWN02_18012023_24_39.jpg | 0 | 0 | 0 | 0 | No | 0.00 |
| Isa_ISCWN02_18012023_24_40.jpg | 0 | 0 | 0 | 0 | No | 0.00 |
| Isa_ISCWN02_18012023_26_27.jpg | 0 | 0 | 0 | 0 | No | 0.00 |
| Isa_ISCWN02_18012023_27_28.jpg | 0 | 0 | 0 | 0 | No | 0.00 |
| Isa_ISCWN02_18012023_28_32.jpg | 0 | 0 | 0 | 0 | No | 0.00 |
| Isa_ISCWN02_18012023_34_25.jpg | 0 | 0 | 0 | 0 | No | 0.00 |
| Isa_ISCWN02_18012023_38_23.jpg | 0 | 0 | 0 | 0 | No | 0.00 |
| Isa_ISCWN02_18012023_42_22.jpg | 0 | 0 | 0 | 0 | No | 0.00 |
| Isa_ISCWN02_18012023_44_25.jpg | 0 | 0 | 0 | 0 | No | 0.00 |
| Isa_ISCWN02_18012023_51_23.jpg | 0 | 0 | 0 | 0 | No | 0.00 |
| Isa_ISCWN02_18012023_55_22.jpg | 0 | 0 | 0 | 0 | No | 0.00 |
| Isa_ISCWN02_18012023_6_62.jpg | 0 | 0 | 0 | 0 | No | 0.00 |
| Isa_ISCWN02_18012023_62_16.jpg | 0 | 0 | 0 | 0 | No | 0.00 |
| Isa_ISCWN02_18012023_63_18.jpg | 0 | 0 | 0 | 0 | No | 0.00 |
| Isa_ISCWN02_18012023_67_20.jpg | 0 | 0 | 0 | 0 | No | 0.00 |
| Isa_ISCWN02_18012023_73_24.jpg | 0 | 0 | 0 | 0 | No | 0.00 |
| Isa_ISCWN02_18012023_75_34.jpg | 0 | 0 | 0 | 0 | No | 0.00 |
| Isa_ISCWN02_18012023_78_36.jpg | 0 | 0 | 0 | 0 | No | 0.00 |
| Isa_ISCWN02_18012023_79_42.jpg | 0 | 0 | 0 | 0 | No | 0.00 |
| Isa_ISCWN02_18012023_81_45.jpg | 0 | 0 | 0 | 0 | No | 0.00 |
| Isa_ISCWN02_18012023_87_42.jpg | 0 | 0 | 0 | 0 | No | 0.00 |
| Isa_ISCWN02_18012023_87_46.jpg | 0 | 0 | 0 | 0 | No | 0.00 |
| Isa_ISCWN02_18012023_9_52.jpg | 0 | 0 | 0 | 0 | No | 0.00 |
| Isa_ISCWN02_18012023_94_49.jpg | 0 | 0 | 0 | 0 | No | 0.00 |
| Isa_ISCWN02_18012023_99_38.jpg | 0 | 0 | 0 | 0 | No | 0.00 |
| Isa_ISTI01-02-03_27012023_10_32.jpg | 0 | 0 | 0 | 0 | No | 0.00 |
| Isa_ISTI01-02-03_27012023_12_48.jpg | 0 | 0 | 0 | 0 | No | 0.00 |
| Isa_ISTI01-02-03_27012023_12_51.jpg | 0 | 0 | 0 | 0 | No | 0.00 |
| Isa_ISTI01-02-03_27012023_14_7.jpg | 0 | 0 | 0 | 0 | No | 0.00 |
| Isa_ISTI01-02-03_27012023_15_35.jpg | 0 | 0 | 0 | 0 | No | 0.00 |
| Isa_ISTI01-02-03_27012023_18_22.jpg | 0 | 0 | 0 | 0 | No | 0.00 |
| Isa_ISTI01-02-03_27012023_19_15.jpg | 0 | 0 | 0 | 0 | No | 0.00 |
| Isa_ISTI01-02-03_27012023_20_20.jpg | 0 | 0 | 0 | 0 | No | 0.00 |
| Isa_ISTI01-02-03_27012023_20_27.jpg | 0 | 0 | 0 | 0 | No | 0.00 |
| Isa_ISTI01-02-03_27012023_20_29.jpg | 0 | 0 | 0 | 0 | No | 0.00 |
| Isa_ISTI01-02-03_27012023_23_21.jpg | 0 | 0 | 0 | 0 | No | 0.00 |
| Isa_ISTI01-02-03_27012023_23_3.jpg | 0 | 0 | 0 | 0 | No | 0.00 |
| Isa_ISTI01-02-03_27012023_23_5.jpg | 0 | 0 | 0 | 0 | No | 0.00 |
| Isa_ISTI01-02-03_27012023_25_7.jpg | 0 | 0 | 0 | 0 | No | 0.00 |
| Isa_ISTI01-02-03_27012023_26_20.jpg | 0 | 0 | 0 | 0 | No | 0.00 |
| Isa_ISTI01-02-03_27012023_29_9.jpg | 0 | 0 | 0 | 0 | No | 0.00 |
| Isa_ISTI01-02-03_27012023_30_11.jpg | 0 | 0 | 0 | 0 | No | 0.00 |
| Isa_ISTI01-02-03_27012023_30_8.jpg | 0 | 0 | 0 | 0 | No | 0.00 |
| Isa_ISTI01-02-03_27012023_31_12.jpg | 0 | 0 | 0 | 0 | No | 0.00 |
| Isa_ISTI01-02-03_27012023_32_11.jpg | 0 | 0 | 0 | 0 | No | 0.00 |
| Isa_ISTI01-02-03_27012023_32_19.jpg | 0 | 0 | 0 | 0 | No | 0.00 |
| Isa_ISTI01-02-03_27012023_36_12.jpg | 0 | 0 | 0 | 0 | No | 0.00 |
| Isa_ISTI01-02-03_27012023_37_31.jpg | 0 | 0 | 0 | 0 | No | 0.00 |
| Isa_ISTI01-02-03_27012023_38_11.jpg | 0 | 0 | 0 | 0 | No | 0.00 |
| Isa_ISTI01-02-03_27012023_38_31.jpg | 0 | 0 | 0 | 0 | No | 0.00 |
| Isa_ISTI01-02-03_27012023_38_32.jpg | 0 | 0 | 0 | 0 | No | 0.00 |
| Isa_ISTI01-02-03_27012023_39_13.jpg | 0 | 0 | 0 | 0 | No | 0.00 |
| Isa_ISTI01-02-03_27012023_41_31.jpg | 0 | 0 | 0 | 0 | No | 0.00 |
| Isa_ISTI01-02-03_27012023_46_29.jpg | 0 | 0 | 0 | 0 | No | 0.00 |
| Isa_ISTI01-02-03_27012023_47_33.jpg | 0 | 0 | 0 | 0 | No | 0.00 |
| Isa_ISTI01-02-03_27012023_55_71.jpg | 0 | 0 | 0 | 0 | No | 0.00 |
| Isa_ISTI01-02-03_27012023_55_73.jpg | 0 | 0 | 0 | 0 | No | 0.00 |
| Isa_ISTI01-02-03_27012023_56_36.jpg | 0 | 0 | 0 | 0 | No | 0.00 |
| Isa_ISTI01-02-03_27012023_57_50.jpg | 0 | 0 | 0 | 0 | No | 0.00 |
| Isa_ISTI01-02-03_27012023_58_66.jpg | 0 | 0 | 0 | 0 | No | 0.00 |
| Isa_ISTI01-02-03_27012023_58_68.jpg | 0 | 0 | 0 | 0 | No | 0.00 |
| Isa_ISTI01-02-03_27012023_60_59.jpg | 0 | 0 | 0 | 0 | No | 0.00 |
| Isa_ISTI01-02-03_27012023_61_53.jpg | 0 | 0 | 0 | 0 | No | 0.00 |
| Isa_ISTI01-02-03_27012023_61_59.jpg | 0 | 0 | 0 | 0 | No | 0.00 |
| Isa_ISTI01-02-03_27012023_64_48.jpg | 0 | 0 | 0 | 0 | No | 0.00 |
| Isa_ISTI01-02-03_27012023_66_38.jpg | 0 | 0 | 0 | 0 | No | 0.00 |
| Isa_ISTI01-02-03_27012023_67_48.jpg | 0 | 0 | 0 | 0 | No | 0.00 |
| Isa_ISTI01-02-03_27012023_68_41.jpg | 0 | 0 | 0 | 0 | No | 0.00 |
| Isa_ISVB01_27012023_1_10.jpg | 0 | 0 | 0 | 0 | No | 0.00 |
| Isa_ISVB01_27012023_11_6.jpg | 0 | 0 | 0 | 0 | No | 0.00 |
| Isa_ISVB01_27012023_12_11.jpg | 0 | 0 | 0 | 0 | No | 0.00 |
| Isa_ISVB01_27012023_12_6.jpg | 0 | 0 | 0 | 0 | No | 0.00 |
| Isa_ISVB01_27012023_2_6.jpg | 0 | 0 | 0 | 0 | No | 0.00 |
| Isa_ISVB01_27012023_20_8.jpg | 0 | 0 | 0 | 0 | No | 0.00 |
| Isa_ISVB01_27012023_20_9.jpg | 0 | 0 | 0 | 0 | No | 0.00 |
| Isa_ISVB01_27012023_21_9.jpg | 0 | 0 | 0 | 0 | No | 0.00 |
| Isa_ISVB01_27012023_3_10.jpg | 0 | 0 | 0 | 0 | No | 0.00 |
| Isa_ISVB01_27012023_40_17.jpg | 0 | 0 | 0 | 0 | No | 0.00 |
| Isa_ISVB01_27012023_43_20.jpg | 0 | 0 | 0 | 0 | No | 0.00 |
| Isa_ISVB01_27012023_45_32.jpg | 0 | 0 | 0 | 0 | No | 0.00 |
| Isa_ISVB01_27012023_45_33.jpg | 0 | 0 | 0 | 0 | No | 0.00 |
| Isa_ISVB01_27012023_45_35.jpg | 0 | 0 | 0 | 0 | No | 0.00 |
| Isa_ISVB01_27012023_47_35.jpg | 0 | 0 | 0 | 0 | No | 0.00 |
| Isa_ISVB01_27012023_47_39.jpg | 0 | 0 | 0 | 0 | No | 0.00 |
| Isa_ISVB01_27012023_47_40.jpg | 0 | 0 | 0 | 0 | No | 0.00 |
| Isa_ISVB01_27012023_50_42.jpg | 0 | 0 | 0 | 0 | No | 0.00 |
| Isa_ISVB01_27012023_51_35.jpg | 0 | 0 | 0 | 0 | No | 0.00 |
| Isa_ISVB01_27012023_51_36.jpg | 0 | 0 | 0 | 0 | No | 0.00 |
| Isa_ISVB01_27012023_52_38.jpg | 0 | 0 | 0 | 0 | No | 0.00 |
| Isa_ISVB01_27012023_55_38.jpg | 0 | 0 | 0 | 0 | No | 0.00 |
| Isa_ISVB01_27012023_57_40.jpg | 0 | 0 | 0 | 0 | No | 0.00 |
| Isa_ISVB01_27012023_6_9.jpg | 0 | 0 | 0 | 0 | No | 0.00 |
| Isa_ISVB01_27012023_69_37.jpg | 0 | 0 | 0 | 0 | No | 0.00 |
| Isa_ISVB01_27012023_70_36.jpg | 0 | 0 | 0 | 0 | No | 0.00 |
| Isa_ISVB01_27012023_71_31.jpg | 0 | 0 | 0 | 0 | No | 0.00 |
| Isa_ISVB01_27012023_71_34.jpg | 0 | 0 | 0 | 0 | No | 0.00 |
| Isa_ISVB01_27012023_72_34.jpg | 0 | 0 | 0 | 0 | No | 0.00 |
| Isa_ISVB01_27012023_73_38.jpg | 0 | 0 | 0 | 0 | No | 0.00 |
| Isa_ISVB01_27012023_74_36.jpg | 0 | 0 | 0 | 0 | No | 0.00 |
| Isa_ISVB01_27012023_74_38.jpg | 0 | 0 | 0 | 0 | No | 0.00 |
| Isa_ISVB02_27012023_115.jpg | 0 | 0 | 0 | 0 | No | 0.00 |
| Isa_ISVB02_27012023_116.jpg | 0 | 0 | 0 | 0 | No | 0.00 |
| Isa_ISVB02_27012023_152.jpg | 0 | 0 | 0 | 0 | No | 0.00 |
| Isa_ISVB02_27012023_30.jpg | 0 | 0 | 0 | 0 | No | 0.00 |
| Isa_ISVB02_27012023_44.jpg | 0 | 0 | 0 | 0 | No | 0.00 |
| Isa_ISVB02_27012023_47.jpg | 0 | 0 | 0 | 0 | No | 0.00 |
| Isa_ISVB02_27012023_71.jpg | 0 | 0 | 0 | 0 | No | 0.00 |
| Isa_ISVB02_27012023_77.jpg | 0 | 0 | 0 | 0 | No | 0.00 |
| Isa_ISVB02_27012023_93.jpg | 0 | 0 | 0 | 0 | No | 0.00 |
| Isa_ISVB02_27012023_97.jpg | 0 | 0 | 0 | 0 | No | 0.00 |
| Isa_ISVB03_77012023_26_15.jpg | 0 | 0 | 0 | 0 | No | 0.00 |
| Isa_ISVB03_77012023_26_18.jpg | 0 | 0 | 0 | 0 | No | 0.00 |
| Isa_ISVB03_77012023_26_19.jpg | 0 | 0 | 0 | 0 | No | 0.00 |
| Fer_FCD01-02-03_20122021_11_9.jpg | 1 | 1 | 1 | 1 | Yes | 0.00 |
| Fer_FCD01-02-03_20122021_29_30.jpg | 1 | 1 | 1 | 1 | Yes | 0.00 |
| Fer_FCD01-02-03_20122021_50_31.jpg | 1 | 1 | 1 | 1 | Yes | 0.00 |
| Fer_FCD01-02-03_20122021_51_23.jpg | 1 | 1 | 1 | 1 | Yes | 0.00 |
| Fer_FCD01-02-03_20122021_56_18.jpg | 1 | 1 | 1 | 1 | Yes | 0.00 |
| Fer_FCD01-02-03_20122021_57_18.jpg | 1 | 1 | 1 | 1 | Yes | 0.00 |
| Fer_FCD01-02-03_20122021_65_12.jpg | 1 | 1 | 1 | 1 | Yes | 0.00 |
| Fer_FEA02-03_18122021_71_38.jpg | 1 | 1 | 1 | 1 | Yes | 0.00 |
| Fer_FPE01-06_18122021_3_52.jpg | 1 | 1 | 1 | 1 | Yes | 0.00 |
| Fer_FPE01-06_18122021_35_19.jpg | 1 | 1 | 1 | 1 | Yes | 0.00 |
| Fer_FPE01-06_18122021_43_34.jpg | 1 | 1 | 1 | 1 | Yes | 0.00 |
| Fer_FPE01-06_18122021_45_0.jpg | 1 | 1 | 1 | 1 | Yes | 0.00 |
| Fer_FPE02_18122021_29_23.jpg | 1 | 1 | 1 | 1 | Yes | 0.00 |
| Fer_FPE03-04-05_18122021_116_10.jpg | 1 | 1 | 1 | 1 | Yes | 0.00 |
| Fer_FPE03-04-05_18122021_22_16.jpg | 1 | 1 | 1 | 1 | Yes | 0.00 |
| Fer_FPM01-02_20012023_22_125.jpg | 1 | 1 | 1 | 1 | Yes | 0.00 |
| Fer_FPM01-02_20012023_3_124.jpg | 1 | 1 | 1 | 1 | Yes | 0.00 |
| Fer_FPM01-02_20012023_43_9.jpg | 1 | 1 | 1 | 1 | Yes | 0.00 |
| Fer_FPM01-02_20012023_46_44.jpg | 1 | 1 | 1 | 1 | Yes | 0.00 |
| Fer_FPM01-02_20012023_53_69.jpg | 1 | 1 | 1 | 1 | Yes | 0.00 |
| Isa_ISTI01-02-03_27012023_20_19.jpg | 1 | 1 | 1 | 1 | Yes | 0.00 |
| Isa_ISTI01-02-03_27012023_57_49.jpg | 1 | 1 | 1 | 1 | Yes | 0.00 |
| Isa_ISTI01-02-03_27012023_59_55.jpg | 1 | 1 | 1 | 1 | Yes | 0.00 |
| Isa_ISVB01_27012023_7_3.jpg | 1 | 1 | 1 | 1 | Yes | 0.00 |
| Isa_ISVB01_27012023_72_37.jpg | 1 | 1 | 1 | 1 | Yes | 0.00 |
| Isa_ISVB02_27012023_154.jpg | 1 | 1 | 1 | 1 | Yes | 0.00 |
| Isa_ISVB03_77012023_27_16.jpg | 1 | 1 | 1 | 1 | Yes | 0.00 |
| Fer_FCD01-02-03_20122021_25_28.jpg | 2 | 2 | 2 | 2 | Yes | 0.00 |
| Fer_FPE01-06_18122021_41_29.jpg | 2 | 2 | 2 | 2 | Yes | 0.00 |
| Fer_FPM01-02_20012023_49_11.jpg | 2 | 2 | 2 | 2 | Yes | 0.00 |
| Fer_FPM01-02_20012023_50_87.jpg | 2 | 2 | 2 | 2 | Yes | 0.00 |
| Fer_FPM01-02_20012023_54_74.jpg | 2 | 2 | 2 | 2 | Yes | 0.00 |
| Fer_FPM01-02_20012023_55_69.jpg | 2 | 2 | 2 | 2 | Yes | 0.00 |
| Fer_FPM01-02_20012023_58_21.jpg | 2 | 2 | 2 | 2 | Yes | 0.00 |
| Fer_FPE01-06_18122021_37_21.jpg | 3 | 3 | 3 | 3 | Yes | 0.00 |
| Fer_FPE02_18122021_2_12.jpg | 3 | 3 | 3 | 3 | Yes | 0.00 |
| Fer_FPM01-02_20012023_0_120.jpg | 3 | 3 | 3 | 3 | Yes | 0.00 |
| Fer_FPM01-02_20012023_25_131.jpg | 3 | 3 | 3 | 3 | Yes | 0.00 |
| Fer_FPM01-02_20012023_49_72.jpg | 3 | 3 | 3 | 3 | Yes | 0.00 |
| Fer_FPM01-02_20012023_51_86.jpg | 3 | 3 | 3 | 3 | Yes | 0.00 |
| Fer_FPM01-02_20012023_8_123.jpg | 3 | 3 | 3 | 3 | Yes | 0.00 |
| Fer_FPM01-02_20012023_10_125.jpg | 4 | 4 | 4 | 4 | Yes | 0.00 |
| Fer_FCD01-02-03_20122021_29_17.jpg | 5 | 5 | 5 | 5 | Yes | 0.00 |
| Fer_FPE02_18122021_11_13.jpg | 6 | 6 | 6 | 6 | Yes | 0.00 |
| Fer_FPE01-06_18122021_57_35.jpg | 7 | 7 | 7 | 7 | Yes | 0.00 |
| Fer_FCD01-02-03_20122021_33_21.jpg | 8 | 8 | 8 | 8 | Yes | 0.00 |
| Fer_FCD01-02-03_20122021_35_42.jpg | 13 | 13 | 13 | 13 | Yes | 0.00 |

# Table S7. Comparison of three experts' counts to assess their similarity in a dataset of three orthomosaics. We calculated the coefficient of variation (CV) among total counts to compare variability across datasets: CV = (Standard deviation / Mean) × 100.

| Orthomosaic name | Expert 1 | Expert 2 | Expert 3 | CV (%) |
| --- | --- | --- | --- | --- |
| Fer_FPE09_18122021 | 97 | 88 | 83 | 7.94 |
| Scris_SRL12_10012021 | 57 | 63 | 54 | 7.90 |
| Scruz_SCM01_06012023 | 80 | 76 | 70 | 6.68 |

**Text S1. Further explanation about the steps used during the process of photogrammetry.**

Photogrammetry involves the orientation, alignment and identification of common features (tie points) across overlapping images collected from an object or a surveyed area. Photogrammetry then entails the generation of a dense point cloud, which is created by triangulating the 3D positions of the identified shared points (step recommended for orthomosaics or 2D models and mandatory for 3D models), then a mesh of the object or area, which connects these points to form a volume or surface, is created, and finally a texture is added, which uses the original image information (pixels) to create a photorealistic 3D model.

After this, an orthomosaic (2D map) can be generated as a GeoTIFF file, which is corrected for size and geographic position using the geographic coordinates embedded within aerial images. Some software (e.g. Pix4D) uses a Digital Surface model (DSM) to create the orthomosaic. A DSM is a three-dimensional representation of the terrain’s surface, including all its features, and is created from the point cloud. The aerial images are then projected into the DSM, rather than being added as a texture, as explained above.

Every photogrammetry software follows a similar process to generate the models, and researchers should investigate their differences when selecting one. Default parameters should be tested first, and adjusted later if needs are not initially met.
